# Pine root exudate terpenoids associate with *Mycobacterium* sp. under summer drought in a mixed Mediterranean forest

**DOI:** 10.1186/s40793-026-00921-8

**Published:** 2026-08-03

**Authors:** Sophie Obersteiner, Yaara Oppenheimer-Shaanan, David Yalin, Dagan Sade, Vered Zavaro, Ziv Reich, Tamir Klein

**Affiliations:** 1 Plant and Environmental Sciences Department, Weizmann Institute of Science, 7610001 Rehovot, Israel; 2 Department of Life Sciences, Achva Academic College, 7980400 Arugot, Israel; 3 Institute of Soil, Water and Environmental Sciences, Volcani Center, ARO, 7505101 Rishon Lezion, Israel; 4 Department of Biomolecular Sciences, Weizmann Institute of Science, 7610001 Rehovot, Israel

**Keywords:** Root exudation, Root exudate metabolites, Rain-exclusion treatment, Soil and rhizosphere bacterial communities, Root-bacteria exchange, Mediterranean forest

## Abstract

**Background:**

Interactions between tree root exudates and rhizosphere bacteria are a key pathway influencing tree health in the forest. However, how this interaction occurs at the chemical level belowground in forest soil still remains unclear. We conducted a 2-year field experiment in a mixed Mediterranean forest with mature trees of *Pinus halepensis*, *Quercus calliprinos* and *Pistacia lentiscus* to examine the interplay between tree roots and bacterial community dynamics under seasonal drought*.* Root exudate rate and metabolite composition were analyzed simultaneously with 16S rRNA sequencing to the species level of soil and root bacterial communities across four seasons.

**Results:**

Root exudates increased, on average, 3.3-fold in the dry vs. wet season across the three species, reaching up to 41.7 μg C day^−1^ cm^−2^. Eighty-nine metabolites were responsive to seasonal drought, consisting largely of amino acids (24), phenolics (21), carbohydrates (11) and terpenoids (9). Across all tree species, eight metabolites were drought-responsive. Metabolite composition varied with tree species and season. However, root bacterial communities changed with host tree species but not with season. Multiple bacterial taxa correlated either positively or negatively with drought-responsive metabolites; for example, 19 actinobacteria species were associated with compounds such as the terpenoid glaucocalyxin A and the carbohydrates deoxyribose and a C5 sugar alcohol, illustrating diverse bacterial responses to seasonal drought-induced exudation shifts.

**Conclusion:**

Our results suggest that in a Mediterranean forest experiencing recurrent seasonal drought, drought-induced shifts in root exudate composition may act as metabolic cues that are associated with changes in the rhizosphere bacterial community, while preserving its overall taxonomic stability.

**Supplementary Information:**

The online version contains supplementary material available at https://doi.org/10.1186/s40793-026-00921-8.

## Introduction

The rate and composition of root exudates released by trees determine their fate in the soil and represent a crucial factor in long-term carbon (C) sequestration in forests, potentially by influencing microbial communities [14, 87]. Soil C sequestration mediated by plant root exudates can contribute to the net removal of CO_2_ from the atmosphere [60]. Root exudates are secreted into the rhizosphere, the hotspot of belowground C processes and the interface where tree roots interact with soil microorganisms. These microorganisms are an integral part of the tree’s belowground C sink, influencing whether C remains stored in the soil as organic C or is respired back to the atmosphere. Beyond their role in C cycling, some microorganisms also enhance plant fitness [59]. Root exudation accounts for approximately 5–21% of plant photosynthates [15, 24, 31, 33]. However, the rate and chemical composition of exudates vary considerably across space and time, influenced by plant species, developmental stage, root traits, nutrient availability, soil type, and biotic and abiotic factors such as for example drought [35, 55, 73, 90].

Drought, a major abiotic stress expected to intensify under climate change, alters both the rate and chemical profile of root exudates. While some studies report a downregulation of exudation under drought [37, 46, 47, 65, 78], a larger body of evidence points to an upregulation in trees [35, 40, 48, 52, 64, 91], suggesting that drought severity, duration, and species-specific C allocation strategies may shape the direction of this response. For instance, Jakoby et al. [35] documented elevated exudation rates in a Mediterranean forest during the dry season, even when photosynthetic C assimilation reached its minimum. Similarly, observations from a temperate forest showed that C allocation to exudates persisted despite drought [11]. Root exudation is often discussed as an active C investment to mobilize nutrients and recruit beneficial microbes, thereby enhancing stress tolerance [46, 47, 59]. Alternatively, the surplus-C theory proposes that exudation may increase when drought constrains growth more strongly than C supply, creating a temporary imbalance between C assimilation and sink demand, particularly at drought onset or under moderate stress [66]. In contrast, exudation may decline under severe or prolonged drought, when photosynthesis and C availability are strongly reduced, soil water content becomes extremely low, or roots lose hydraulic contact with the soil [46, 47, 88]. Further, increasing drought severity can also alter exudate composition: in oak and cypress, secondary metabolites rose to up to about 70% of total exudates under drought, compared with only 15–20% under control conditions [30, 59]. A recent meta-analysis spanning farmland and forest ecosystems further showed drought-induced increases in acetic and citric acids, although overall C exudation rates remained unaffected [93].

While plants shape their microbiomes in part through the chemical composition of root exudates [8, 9, 94], increasing evidence shows that plant–microbe interactions in the rhizosphere operate as two-way exchanges rather than one-sided processes, with microbes associated with changes in plant exudation and metabolism in return [27, 44, 59]. Systemically induced root exudation of metabolites (SIREM) demonstrates that distinct microbial communities can trigger specific systemic changes in plant exudation [44]. In trees under drought stress, bacterial associates can further amplify exudation rates and modify metabolite profiles, highlighting feedback loops that couple plant stress physiology with microbial recruitment and function [59]. The strength and direction of this coupling are species- and context-dependent [38]. In a tropical dry forest, soil microbial communities remained seasonally stable, plausibly buffered by relatively stable soil properties and nutrient cycling [77]. In a switchgrass system, rhizosphere microbiome composition persisted despite shifts in rhizosphere metabolites under short-term drought, suggesting partial decoupling of microbial responses from plant exudation. By contrast, in the same experiment, bean plants recruited specific bacterial taxa under drought, underscoring the species-specific nature of plant–microbe interactions [5]. Additional evidence from wheat showed increased abundance of *Streptomyces coeruleorubidus* and *Leifsonia shinshuensis* under drought, together with higher 4-oxoproline levels in the rhizosphere; 4-oxoproline was further shown to promote *S. coeruleorubidus* growth in vitro, suggesting a role in drought-associated microbial enrichment [46, 47]. Inoculation with these drought-enriched strains improved wheat biomass under drought, particularly root biomass, highlighting how drought-induced microbiome–metabolite interactions can improve plant adaptation [46, 47]. Together, these findings indicate that coupling between biochemical composition and the microbiome varies across environments, timescales and hosts.

The interaction between root exudates and root-associated microbiomes under drought conditions remains poorly understood [17]. Moreover, most existing studies have been conducted under synthetic or controlled conditions using crop and model plant species. To better predict future global C cycling, it is critical to investigate these interactions in natural field settings, particularly in forests, where root exudates influence long-term C sequestration [14].

In this study, we set out to decipher the seasonal dynamics of root exudation rates and composition, as well as the associated soil and rhizosphere bacterial communities, in a mixed Mediterranean forest. We focused on three dominant Mediterranean forest tree species: *Pinus halepensis*, *Quercus calliprinos*, and *Pistacia lentiscus*. Specifically, our objectives were to: (1) characterize seasonal fluctuations in root exudation rate and composition; (2) assess seasonal shifts in soil and rhizosphere bacterial community compositions; and (3) explore correlations between metabolites and bacterial taxa. We hypothesized that: (1) seasonal drought would enhance root exudation rates and shift the metabolic profile toward stress-related metabolites; (2) bacterial communities would display seasonal variation, due to changing root exudates, with stronger effects in the rhizosphere compared to bulk soil; and (3) specific bacterial groups increase, while other decrease, in response to specific exudate metabolite groups, regardless of tree species or season.

## Material and methods

### Site and study design

**Fig. 1:**
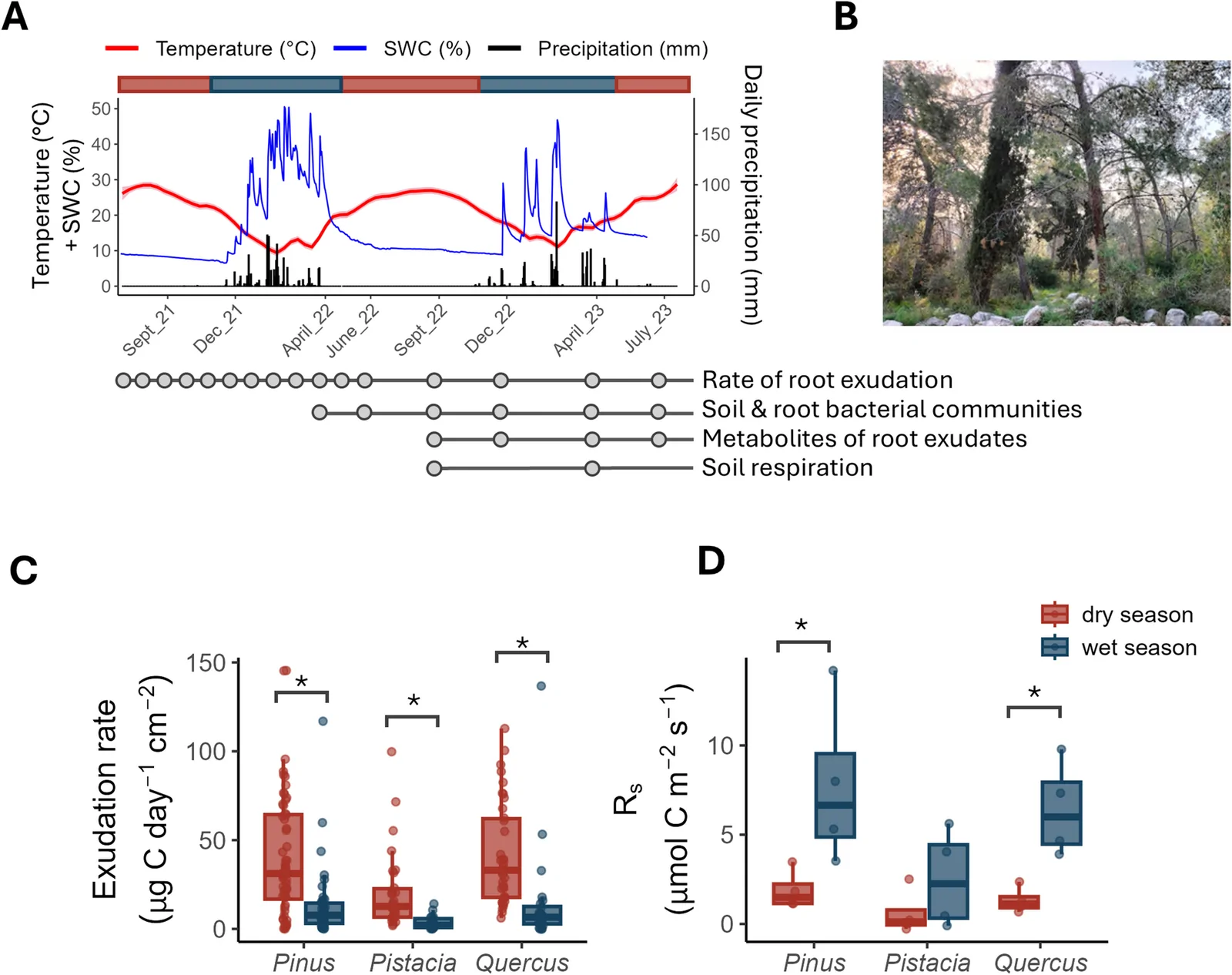
Daily mean temperature (°C), soil water content (SWC; %) at 20 cm depth, daily precipitation (mm), and sampling times for root exudation rate, bacterial communities, root exudate metabolites and soil respiration (**A**) at the mixed Mediterranean forest study site (**B**). Root exudation rate (μg C day^−1^ cm^−2^; sample sizes in Table S2;** C**) and soil respiration (R_s_, μmol C m^−2^ s^−1^; n = 4; **D**) measured beneath *Pinus*, *Pistacia* and *Quercus* in the dry (May–November) and wet (December–April) season. Lines in boxes represent the median, the top and bottom of boxes represent first and third quartiles and whiskers represent the 1.5 interquartile range. Asterisks indicate significant differences between dry and wet seasons (*p* < 0.05)

Field measurements were conducted in a forest research site in a ~ 650 ha mixed Mediterranean forest in the Judean foothills, 4 km south-west of Beit Shemesh, Israel [26, 74, 92]. Twenty-year average annual precipitation is 460 mm, falling mainly between October and May, subjecting the trees to a prolonged drought between May to October. The ten-year mean annual temperature is 20.5 ± 6.9 °C, with August being the hottest month (27.2 ± 0.7 °C) and January being the coldest month (12.4 ± 1.4 °C). Climatic conditions during the study period are shown in Fig. 1A. The forest biomass is dominated by the planted native gymnosperm species *Pinus halepensis* (20.9 ton ha^−1^) and *Cupressus sempervirens* (32.7 ton ha^−1^), as well as the local Mediterranean angiosperm species *Quercus calliprinos* (2.6 ton ha^−1^) and *Pistacia lentiscus* (4.8 ton ha^−1^), considering all individuals with height > 1 m [74] (Fig. 1B). Stem diameter and height of the studied trees are shown in Table S1. The current forest is composed of a combination of planted and local trees (both native). Sixty years ago, it was an overgrazed Mediterranean maquis, with stunted broadleaf vegetation. The canopy tree species, *Pinus halepensis*, was planted in the site ~ 55 years ago, as confirmed by tree-ring observations. The subcanopy species *Quercus calliprinos* and understory species *Pistacia lentiscus* resprouted shortly after, making all studied trees 50–55 years old. The predominant soil type in the study area is Ustolls (Mollisols) [34], characterized by A and C horizons. The A horizon has an average depth of ~ 21 cm, varying between 16 and 25 cm depth. The C horizon extends into the cracks between the weathered limestone bedrock [75].

Six research plots (~ 500 m^2^ each) were established within the forest research site, encompassing the four co-occurring woody species. The plots were assigned to two treatments: three plots subjected to a rainfall reduction treatment to simulate drought conditions, and three control plots receiving ambient precipitation (Fig. S1A). The rainfall reduction was established in November 2021, prior to the first seasonal rains, using an open-pipe system designed to divert approximately 50% of precipitation. Root exudation rate, bacterial communities, root exudation metabolites and soil respiration were measured between July 2021 and July 2023 according to Fig. 1A and Table S3. The rain-exclusion treatment had no significant effect (or only low explanatory contribution) on root exudation rate, metabolite composition, bacterial community composition or soil respiration. Therefore, for visualization purposes, control and rain-exclusion plots were combined in the figures, although rain-exclusion treatment was retained as a factor in the statistical models. Given the drought-adapted tree species and the relatively short manipulation period, near-term treatment effects were not expected. This aligns with concurrent measurements in the same plots showing limited treatment effects on leaf water potential, C assimilation, and transpiration across tree species and seasons, while hyperspectral-based machine learning still distinguished treatments [92]. This suggests that hyperspectral shifts may precede detectable changes in root exudates or rhizosphere bacterial communities.

Climatic conditions were retrieved from continuous measurements recorded at the Israel Meteorological Service Beit Jimal station (~ 750 m away; Fig. 1A).

### Soil measurements

Soil moisture sensors (EC-5; METER, Pullman, WA, USA) were installed beneath the canopy at 20 cm soil depth and connected to ZL6 data loggers. Sensors were logged every 15 min and aggregated to hourly means for analysis. Due to occasional technical issues and animal interference, we retained the mean of three sensors. Short gaps bounded by observations were linearly interpolated; leading/trailing gaps were left as missing.

Soil texture analysis was performed on three samples collected from open areas within the plots, using PARIO (Meter, Munich, Germany) following the manufacturer’s protocol, resulting in 18.67 ± 0.67% sand, 48.33 ± 0.67% silt, and 33.33 ± 0.88% clay. Soil chemistry was assessed using a standardized analysis protocol as described in Rapaport et al. [68]. Soil samples were collected in December 2022 beneath *Pinus*, *Pistacia* and *Quercus* in control and rain-exclusion plots (n = 3) at 10–20 cm depth, air-dried, and sieved through a 2 mm mesh. For water-extractable measurements, 8 g of the < 2 mm soil fraction were mixed with 40 mL deionized water, shaken overnight, and filtered. For Olsen phosphorus levels, 2.5 g of < 2 mm soil fraction were extracted with 50 mL of 0.5 M sodium bicarbonate, shaken for 30 min, and filtered. pH and mineral concentrations (chloride, magnesium, nitrate, ammonium and phosphorus) were quantified using a pH meter (Orion Lab Star pH 11) and a Gallery™ Plus Discrete Analyzer (Thermo Fisher; see results in Table S4).

Continuous soil respiration was measured for ≥ 24 h adjacent to the same *Pinus*, *Pistacia*, and *Quercus* individuals used for root exudation sampling during the dry season (September 2022) and wet season (April 2023), in two plots per treatment (rain-exclusion and control). Collars were installed in the ground at a distance of 0.5–1.0 m from the tree trunk at locations without visible recent soil disturbance. Tree size is provided in Fig. S1. Soil flux measurements were performed using an infrared gas analyzer (IRGA) based LICOR 8100A (LICOR Inc., USA) system equipped with a multiplexer and powered with 12 V rechargeable batteries. Automated opaque cells (LICOR 8100-104) were installed and were closed for 2 min every 30 min. Flux values were calculated with Soil Flux Pro V4.2.1 using the linear regression option. Only flux values with regression R^2^ > 0.5 were used and gap filling was performed up to 3 h of missing data. Each chamber was equipped with a thermistor probe (Licor 8150-203) pressed directly to a depth of ~ 5 cm into the soil adjacent to the chamber.

### Root exudation collection

Root exudates were collected from intact fine roots of *Pinus*, *Pistacia* and *Quercus* along 16 campaigns (Table S3) using a modified non-soil syringe system, based on Phillips et al. [62] and Jakoby et al. [35]. We prioritized resampling the same focal trees, but when necessary incorporated additional trees of the same species from the same plot to reduce oversampling and allow recovery between sampling events. We sampled two to three roots per tree (subsamples) and three trees per treatment × species combination (biological replicates). Because root systems are intertwined in the mixed Mediterranean forest, accurate species assignment of excavated roots during sampling is challenging. To ensure correct identification while avoiding disturbance from excavating and tracing roots to stems, we verified root species via DNA barcoding (see Sect. Validation of tree species identity). Samples that failed verification were excluded from further analysis, and final biological replicate counts are reported in Table S2. Fine roots were sampled from the 5–25 cm depth of the A horizon and remained attached to the tree during the entire procedure until harvest. Intact fine roots were gently rinsed with C-free nutrient solution (0.5 mM NH_4_NO_3_, 0.1 mM KH_2_PO_4_, 0.2 mM K_2_SO_4_, 0.2 mM MgSO_4_, 0.3 mM CaCl_2_) and placed back into the soil to allow recovery from disturbance for ~ 24 h. Next, the roots were cleansed again and placed into 25 mL plastic syringes filled with 0.5–1.3 mm acid-washed glass beads and 10 mL of the C-free nutrient solution (water content in syringes was ~ 21%). In a previous study, we showed that this water content increased root exudation compared with lower water content in syringes; therefore, absolute exudation rates may be shifted upward, but the relative treatment patterns are expected to remain comparable [58]. To block light and prevent heat exposure, the syringes were wrapped in aluminum foil and covered with soil. Incubations proceeded for 48 h, following the recommended incubation time for our Mediterranean forest soils [58], after which the nutrient solution was collected. To ensure representative C recovery, each syringe was then flushed with an additional 10 mL double-distilled water. For each measuring campaign, 3–4 blanks were also prepared: blank syringes (without roots) underwent the same procedure to account for potential contamination, and any C detected in blank syringes was subtracted from the samples. Following incubation, the two replicate solutions were divided into two aliquots: one for determining exudation rates and the other for metabolomic analysis. All samples were filtered through 0.22 μm sterile syringe filters (Millex PVDF, Millipore Co., Billerica, MA, USA); samples for exudation rate analysis were stored at −20 °C, while samples for metabolomic analysis were stored at −80 °C until analysis.

### Organic C analysis of root exudation

Total organic carbon (TOC) content was quantified using a total organic carbon analyzer (Aurora 1030 W TOC Analyzer with a 1088 rotary TOC auto sampler; OI Analytical, TX, USA). Root exudation rate was calculated as the total C recovered from the pooled root system over the incubation period, normalized by root surface area, and reported as specific exudation rates (μg C cm^−2^ day^−1^). Root exudation rates of subsamples of the same tree were averaged. Root surface area was measured after exudate collection using a flatbed scanner (HP Color Jet Pro MFP M477fdn) and analyzed with RhizoVision Explorer 2.0.3.

### Metabolite analysis of root exudates using LC–MS

Root exudate samples from four campaigns (Sept 2022, Dec 2022, April 2023 and July 2023; n = 1–3), along with one blank sample per campaign (samples without a root), were lyophilized for 72 h and analyzed for polar and semi-polar metabolites using liquid chromatography–mass spectrometry (LC–MS) at MS-Omics (Vedbæk, Denmark). Metabolite samples were re-dissolved in 1 mL methanol:water (1:1, v:v), vortexed, and centrifuged (4 °C, 2 min, 1,000 × rcf). The supernatant was split for polar and semi-polar metabolite profiling, dried under nitrogen gas, and reconstituted in method-specific solvents. A pooled sample was prepared by mixing 60 µL aliquots from each sample.

#### Analysis of polar metabolites

Extracts were re-dissolved in 150 µL acetonitrile:water (1:1 vol/vol) and spiked with isotope-labeled standards before injection. Ultra-high-performance liquid chromatography (UHPLC)-based high-resolution mass spectrometry (HRMS) was performed using a Vanquish UHPLC system (Thermo Fisher Scientific) coupled to a Q Exactive™ HF Hybrid Quadrupole-Orbitrap mass spectrometer with electrospray ionization (ESI). Chromatographic separation employed hydrophilic interaction liquid chromatography (HILIC) on an ACQUITY Premier BEH Amide column (1.7 μm, 2.1 mm × 100 mm, Waters) under a two-step gradient (12 min) followed by a 4 min equilibration (flow rate: 450 μL/min, column temp: 30 °C, injection volume: 2 μL). Mobile phases included eluent A (10 mM ammonium formate in acetonitrile, pH 10) and eluent B (10 mM ammonium formate in 40% acetonitrile, pH 10, with 0.1% medronic acid). Mass spectrometry analysis was performed in both positive and negative ionization modes at a resolution of 120,000 and a scan range of 60–900 m/z, with iterative data-dependent MS/MS (TopN = 10, NCE = 20, 40, 60). The pooled QC sample was analyzed in MS/MS fragmentation mode for the identification of compounds.

#### Analysis of semi-polar metabolites

Samples were re-dissolved in 120 µL water:methanol:formic acid (89.9:10.0:0.1 vol/vol/vol + ammonium formate 10 mM) and diluted 5 × in mobile phase eluent A (10 mM ammonium formate + 0.1% formic acid in water) and spiked with isotope-labeled standards. Eluent B consisted of 10 mM ammonium formate in methanol with 0.1% formic acid. Separation was performed using ACQUITY UPLC HSS T3 C18 column (2.1 × 150 mm, 1.8 μm; Waters). The samples were analyzed based on a modified protocol of Doneanu et al. [23] using liquid chromatography, coupled with HRMS as described above for analysis of polar metabolites.

For both semi-polar and polar datasets, peaks were aligned, and areas were extracted with Compound Discoverer 3.3 (Thermo Scientific, MA, USA) and Skyline 23.1 [2]. Chromatographic alignment was applied with an adaptive curve (0.4 min max, 4 ppm) and peaks finally grouped across injections (using retention time and accurate mass). Compounds were annotated at four confidence levels: Level 1—retention time (matched to authentic in-house standards), accurate mass (± 3 ppm), and MS/MS spectra; Level 2a—retention time and accurate mass (± 3 ppm; matched to authentic in-house standards); Level 2b—accurate mass and MS/MS spectra (matched to external libraries); Level 3—accurate mass only (matched to external libraries). Overall, 1985 polar and 2731 semi-polar LC–MS features were detected, of which 1243 and 2249, respectively, remained unannotated. For the main statistical analysis, we retained only Level 1 and Level 2a metabolites, as these were matched to authentic in-house standards and allowed reliable biological interpretation. Lower-confidence annotations and unannotated features were excluded because their identities and ecological functions could not be assigned with sufficient confidence, although this may omit biologically relevant but under-characterized compounds. Within each campaign, metabolites were excluded when the mean intensity of experimental samples was equal to or lower than the mean intensity of experimental blanks. Data were normalized using two distinct approaches to reflect the biological context of each parameter. Exudate TOC was expressed per root surface area to standardize the C flux across the root-soil interface. In contrast, individual metabolite peak areas were normalized to root dry weight to represent the internal metabolic concentration relative to total biomass, following standard protocols for plant tissue metabolomics [89, 6].

### Bacterial community analysis

Soil and root samples were collected for bacterial analysis from the same individual trees of *Pinus*, *Pistacia*, and *Quercus* within each plot across six sampling campaigns (April 2022, June 2022, September 2022, December 2022, April 2023, and July 2023). The last four campaigns coincided with metabolite exudation sampling. Soil was sampled 1.5 m from the stem following litter removal, and only live, attached roots were collected. Samples were kept on dry ice in the field and stored at −80 °C until analysis. Genomic DNA was extracted from ~ 100 mg of root tissue and 250 mg of soil using an automated extraction workflow on a Tecan liquid-handling robot with the ZymoBIOMICS™ 96 MagBead DNA Kit (Zymo Research, Cat. No. D4308), following the manufacturer’s protocol. Tree species identity of root samples was verified by Sanger sequencing of the ITS-2 region, as described below (see Sect. *Validation of tree species identity*).

#### Long-read database augmentation (PacBio)

To expand the reference database, full-length 16S rRNA amplicons were generated with Locked Nucleic Acid (LNA) primers (QIAGEN, Cat. No. 339406) and sequenced on the PacBio Vega platform [42]. Pooled amplicons from root and soil samples were converted to SMRTbell libraries (PacBio, Kit 2.0; PN 100-938-900) and processed in SMRT Link for quality control, demultiplexing, and chimera removal. Sequences were clustered at 99% identity using CD-HIT [28] and compared to the SILVA database [67]. Novel sequences (< 99% identity) were added to the reference set, contributing 66,310 full-length 16S sequences. Full details and scripts are available in CoSMIC [42], (GitHub: NoamShental/CoSMIC). Due to the database enrichment, the number of unassigned reads in the samples was reduced by 13.6% after the enrichment (Table S5).

#### Short-read library preparation and sequencing

Short-read amplicon libraries were prepared using a two-step PCR protocol targeting multiple 16S rRNA gene regions (V1–V2, V2–V3, V3–V4, V4–V5, V5–V7, and V7–V9). Primers included Illumina adapters, heterogeneity spacers, and Unique Molecular Identifiers (UMIs) for error correction [42, 69]. First-step PCR reactions (30 µL) contained KAPA HiFi HotStart ReadyMix, primers (0.5 µM), and 20 ng template DNA. Non-overlapping primer pairs were multiplexed within single reactions. Cycling conditions were 95 °C for 2 min, followed by 6 cycles of 95 °C for 30 s, 52 °C for 30 s, and 72 °C for 30 s, with a final extension at 72 °C for 1 min. Amplicons were purified using Qiagen columns and KAPA Pure Beads. In the second PCR, Illumina i5/i7 indices were added (50 µL reactions) and libraries were amplified (16 cycles: 95 °C 30 s, 60 °C 30 s, 72 °C 30 s). Indexed libraries were purified, quantified by qPCR, and size-validated using TapeStation. Libraries were pooled, concentrated, and sequenced on an Illumina NovaSeq platform, yielding 12.5K–1.5M reads per sample.

#### Amplicon processing and analysis

Raw reads were processed using the CoSMIC pipeline [42]. Reads were demultiplexed by variable region with Cutadapt [54], and long nonspecific amplicons were excluded. Paired-end reads were quality-filtered and denoised with DADA2 [12] to generate amplicon sequence variants (ASVs). Bacterial ASVs were taxonomically profiled using SMURF [29, 42] across all six regions, employing the augmented reference database (SILVA plus PacBio-derived full-length sequences).

#### Validation of tree species identity

All roots used for exudation analysis (rate and metabolite composition) and bacterial community analysis were verified for host species identity using the tree ITS-2 region. For roots used in the exudation analysis, DNA was extracted following Obersteiner et al. [58]. Briefly, 3–4 mg of frozen, ground root tissue was combined with 500 μl of 0.5 M NaOH containing 1.5% polyvinylpyrrolidone (PVP). Samples were vortexed, incubated at 60 °C for 10 min, and centrifuged at 20,000 × g for 5 min. The supernatant (2 μl) was transferred into 150 μl of 100 mM Tris–HCl (pH 8.0), mixed, and used for PCR amplification. To verify the host identity of roots sampled for bacterial analysis, DNA extracts (prepared as described in Sect. *Bacterial community analysis*) were likewise used for PCR.

PCR amplifications were performed in 25 μl volumes containing 1.25 units Dream Taq DNA polymerase (Thermo Scientific), 2 μl DNA, and 1 μl each of primers ITS2-S21 (5′-ATGCGATACTTGGTGTGAAT) and ITS2-S3rR (5′-GACGCTTCTCCAGACTACAAT) [50]. Cycling conditions consisted of an initial denaturation at 94 °C for 1 min, followed by 35 cycles of 94 °C for 30 s, 56 °C for 30 s, and 72 °C for 1 min; followed by a final extension at 72 °C for 8 min. PCR products (10 μl) were checked on a 1% agarose gel; samples yielding a single clear band were purified with Exo-1 and rSAP and Sanger-sequenced (Biological Services Department, Weizmann Institute of Science, Rehovot, Israel) unidirectionally with primer ITS2-S3rR. ITS2 sequences were manually compared against the NCBI database using BLAST, and only samples with unambiguous tree species identity were retained for further analysis; samples not matching the targeted host were excluded (see Table S2 for final number of replicates).

### Statistical analysis

Data were analyzed and visualized using R version 4.4.2 [72] and its interface RStudio [71], unless otherwise stated.

#### Exudation rate and soil respiration rate

Exudation rate was analyzed with a linear mixed-effects model (LMM) including fixed effects of season (wet, dry), treatment (control, rain-exclusion), species (*Pinus*, *Pistacia*, *Quercus*), and all interactions, with a random intercept for tree ID to account for repeated measures (Table S6). Models were fit by restricted maximum likelihood using lme4::lmer. Because sample sizes were unequal, we used sum-to-zero contrasts and conducted Type-III tests with car::Anova. Planned simple-effects were estimated with emmeans as the dry vs wet contrast within each species, averaged over treatment with sample-size weighting (weights = “proportional”); *p*-values for contrast families were adjusted with Benjamini–Hochberg. Soil respiration was analyzed identically, except that the design was balanced (n = 4 per season), hence proportional weighting was not used (Table S6).

#### Metabolite analysis

Changes in metabolite composition were visualized as relative peak intensity (row-wise min–max scaled to 0–100%), calculated as the means of log_2_ transformed peak areas of biological replications of tree species × time (n = 5–6) using ComplexHeatmap. Only metabolites that changed significantly in at least one species over time were presented in the heatmap. For each metabolite × species, we fit a linear mixed-effects model using lme4/lmerTest: y ~ time + rain-exclusion treatment + (1 | Plant ID) with time (September 2022, December 2022, April 2023, July 2023) as a fixed effect and Plant ID as a random factor. F-tests of time were Benjamini–Hochberg FDR-adjusted per species and metabolites with *p* < 0.05 were deemed time-responsive. PCA was generated with the complete log_2_ transformed metabolite dataset and results were visualized with 95% ellipses. Significant differences between groups in the PCAs were determined by PERMANOVA using the adonis function within “vegan”. For statistical results for metabolite analysis see Table S7.

#### Bacterial community analysis

Sequences belonging to the Eukaryotic domain and Chloroplast order were removed from the dataset and 3,636 bacterial and archaeal ASVs remained for analysis. Because archaeal ASVs represented only a minor fraction of the dataset (0.25% of ASVs), we refer to the analyzed communities as bacterial communities throughout the manuscript. Eight of the 169 samples had low sequence reads (between 1,000 and 10,000 reads), but were retained due to the already limited sample replication. Phyloseq was used to perform alpha diversity analysis, including Observed, Shannon, and Simpson indices. Statistical significance was assessed using generalized linear mixed models (GLMM), accounting for the effects of species, time, and sample type, with Plant ID as a random effect. Beta diversity was evaluated through Principal Coordinate Analysis (PCoA) on Bray–Curtis dissimilarity matrices, which were calculated after relative abundance normalization. This analysis was performed on the full community as well as soil and root subsets. To assess the effects of sample type, rain-exclusion treatment, species, and time on bacterial community composition, a PERMANOVA was conducted using the Bray–Curtis distance matrix, with Tree ID included as a blocking factor. The adonis2 function was used to perform this analysis (Table S8). For all subsequent analyses, ASVs were filtered for prevalence (≥ 2 reads in at least 10% of samples) and normalized using the median-of-ratios relative log expression (RLE). For the phylum-level abundance analysis of soil and root bacterial communities, data were instead normalized using total sum scaling (TSS) and visualized with the microViz package. Enrichment analysis between soil and root samples was conducted using MaAsLin2, with sample type (root vs. soil) as the primary fixed effect, and time, species, and rain-exclusion treatment as covariates. Phyla with significant log_2_ fold changes (log_2_ FC > 0.8, *p* < 0.05, FDR corrected) were visualized. To identify significant bacterial features in the root bacterial community associated with different species and seasons, Linear Discriminant Analysis (LDA) Effect Size (LEfSe) was used, with an LDA score threshold of > 2 and a *p*-value cutoff of < 0.05 using MicrobiomeAnalyst 2.0 [51] (Table S9). The core bacterial community of root samples was also analyzed using MicrobiomeAnalyst.

#### Correlation matrix of root exudate metabolites and rhizosphere bacterial communities

To assess links between exudates and the rhizosphere bacterial community, we correlated metabolite and bacterial datasets. Due to missing bacterial community data, *Quercus* (July 2023) was excluded. Group means (time × tree species) of log_2_-transformed peak areas for the 89 time-responsive metabolites were correlated with group means (time × tree species) of relative read abundance at the species level (≥ 5% prevalence). Spearman correlations were calculated between group means of metabolites and bacterial species. To reduce matrix complexity, metabolites and species with at least one high absolute correlation (|R|> 0.85) were retained for heatmap visualization. This threshold was used as an exploratory filter to highlight strong association patterns.

For selected metabolite–bacteria correlations from clusters 1E and 3E, values were z-scored within each pair to enable comparability while preserving seasonal structure. Linear relationships were assessed by OLS regression (equation, Spearman ρ, p), with mixed-effects slopes (lme4) included as a robustness check.

## Results

### Rate of root exudates and soil respiration

To assess the seasonal effect on belowground C fluxes, we measured root exudation rates for two years and soil respiration at two time points (September 2022 and April 2023) in *Pinus*, *Pistacia*, and *Quercus*. Our root exudation measurements started in the dry season, July 2021, after a below-average wet season with 388 mm precipitation (long-term mean: 460 mm) followed by two average wet seasons with 505 mm and 521 mm of rain in 2022 and 2023, respectively. SWC ranged from 6.4% in November 2021 up to 54.0% in February 2022 (Fig. 1A). Two years of measurements (July 2021–July 2023) showed that the rate of root exudates varied throughout the seasons in *Pinus*, *Pistacia* and *Quercus*, and root exudation rates increased on average 3.3-fold in the dry compared to the wet season (Figs. 1C, S1B–D). Dry vs. wet season and species exerted a significant effect on root exudation rate (season: F_1, 189_ = 18.71, *p* < 0.001, species: F_2, 14_ = 6.18, *p* = 0.01), whereas rain-exclusion treatment was not found to be significant. In the hot and dry season (May–November) exudation rate was higher by 206%, 497% and 183% compared to the cooler wet season (December–April) in *Pinus*, *Pistacia* and *Quercus*, respectively (Fig. 1C). In the wet season, root exudation reached average levels close to zero. Overall, root exudation rates ranged from 0.0–145.4 μg C day^−1^ cm^−2^, with *Pistacia* having the lowest average rate. Average root exudation in the dry season was 40.6 ± 4.6, 20.7 ± 4, 41.7 ± 4.7 μg C day^−1^ cm^−2^ in *Pinus*, *Pistacia* and *Quercus* respectively (Fig. 1C).

Opposite to root exudation seasonality, soil respiration was higher in the wet season compared to the dry season in all species (Fig. 1D). Season and species had a significant effect on soil respiration (season: F_1, 12_ = 21.9, *p* < 0.001, species: F_2, 12_ = 4.5, *p* = 0.035), while rain-exclusion treatment did not show any significant effect (Fig. S2). Respiration increased in the wet season by 308%, 318% and 386% in *Pinus*, *Pistacia* and *Quercus*, respectively. In the wet season *Pinus* reached the highest respiration values of 7.8 ± 2.3 μmol C m^−2^ s^−1^ while *Pistacia* showed the lowest values in the dry season with 0.6 ± 0.6 μmol C m^−2^ s^−1^.

### Composition of root exudates

**Fig. 2:**
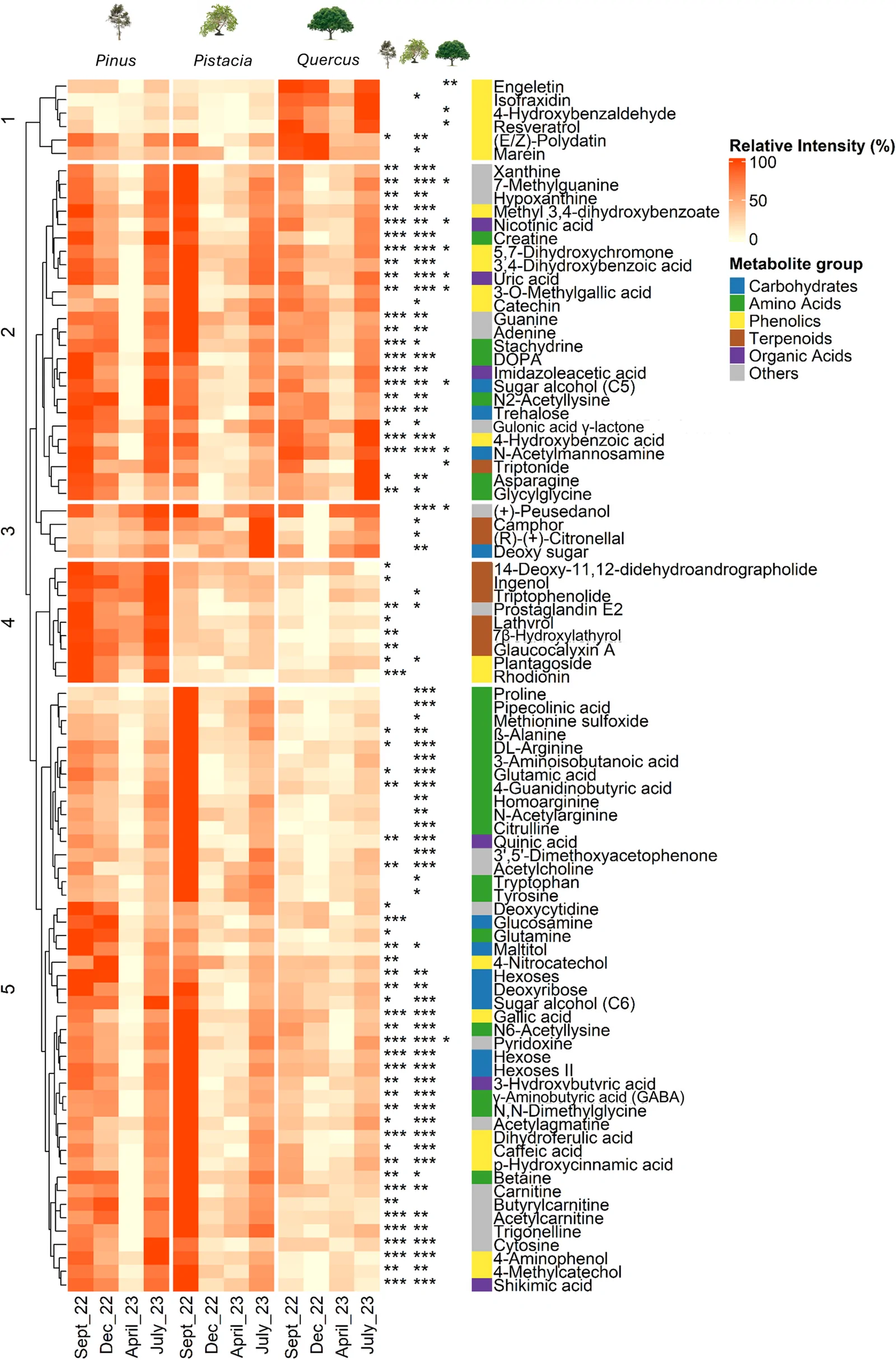
Relative Intensity (%) of root exudate metabolites of *Pinus*, *Pistacia* and *Quercus* in four seasons (Sept 22, Dec 22, April 23 and July 23). Each cell represents the averaged normalized peak height of metabolites (n = 5–6). Only metabolites showing significant temporal changes in at least one species are displayed (linear mixed-effects model with FDR < 0.05). Hierarchical clustering was applied to rows using Euclidean distance. Stars in the right annotation indicate significant effects of time within each tree species (*p* < 0.05 (*), 0.01 (**) and 0.001 (***))

**Fig. 3:**
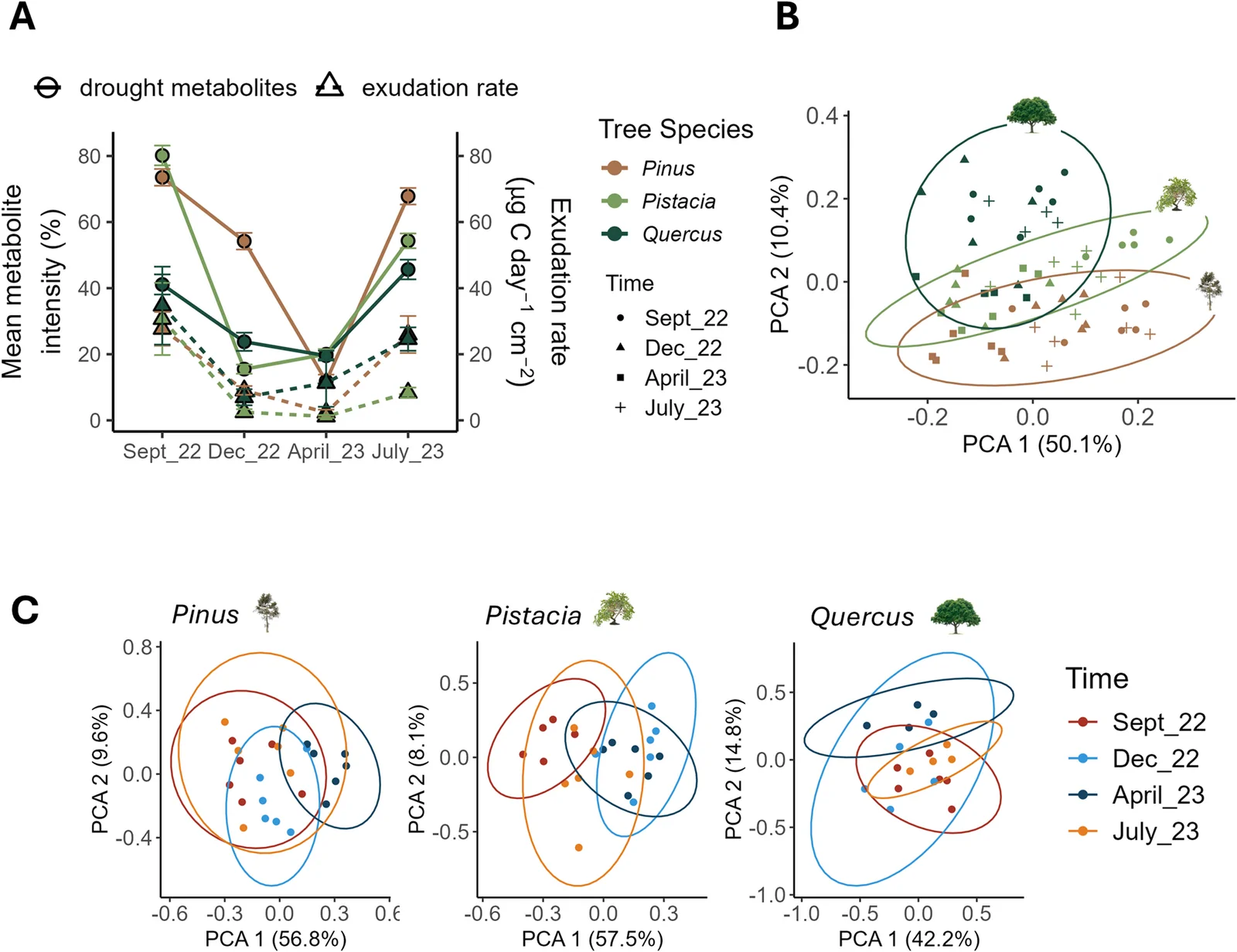
Mean of peak heights of all time-responsive root exudate metabolites (from Fig. 2) and exudation rate for *Pinus*, *Pistacia* and *Quercus* across the four seasons (Sept 22, Dec 22, April 23 and July 23; **A**), time-responsive root metabolites showed significant temporal changes in at least one species (linear mixed-effects model with FDR < 0.05). Error bars in panel A represent ± standard error (SE). PCA of root exudate metabolite profiles of *Pinus*, *Pistacia* and *Quercus* (**B**) and for each tree species at the four seasons (**C**). Ellipses represent 95% confidence intervals

To assess the compositional change of root exudate metabolites throughout the seasons, metabolite profiling based on LC–MS was conducted on root exudate samples for *Pinus*, *Pistacia* and *Quercus* across four time points (September 2022, December 2022, April 2023 and July 2023), resulting in the unequivocal annotation of a total of 196 metabolites: 46 polar metabolites and 150 semi-polar metabolites (certainty levels 1 and 2a; see Metabolite analysis of root exudates using LC–MS). Among these 196 metabolites and after excluding metabolites with levels lower than the blanks, 89 metabolites exhibited significant changes across time in at least one species (Fig. 2). Out of the 89 metabolites, *Pistacia* showed the highest number with 75, followed by *Pinus* with 67, while *Quercus* showed changes in only 13 metabolites. The clustering analysis further revealed species-specific patterns in the root exudate metabolome. Cluster 1, consisting solely of phenolics, exhibited the highest intensities in *Quercus*, with engeletin, 4-hydroxybenzaldehyde, and resveratrol uniquely associated with this species. Cluster 2 included metabolites that showed significant temporal variation across most species. Cluster 3 was characterized by metabolites that exhibited significant time-dependent changes specifically in *Pistacia*, such as camphor and deoxy sugar. Cluster 4, which primarily consisted of terpenoids (14-deoxy-11,12-didehydroandrographolide, ingenol, triptophenolide, lathyrol, 7β-hydroxylathyrol, and glaucocalyxin A), exhibited highest intensities in *Pinus*. Notably, these terpenoids (except triptophenolide) were only responsive to seasonal drought in *Pinus*. Finally, cluster 5, the largest group with 45 metabolites, showed a pronounced peak in *Pistacia* in September. Among these, 18 amino acids, 7 carbohydrates, and 7 phenolics were included, with notable metabolites such as acetylcarnitine and trigonelline. Across species, the mean of relative metabolite intensities followed a consistent seasonal pattern, with highest values in the dry season (September and July) and lowest values in the wet season (December and April). Intensities in *Pinus* were relatively high in December (53.76 ± 2.51%) compared to *Pistacia* and *Quercus*, but then also showed a decline reaching 11.18 ± 1.92% in April. *Pistacia* reached the highest intensity in September (80.53 ± 3.01%) and dropped by almost 80% in December (15.39 ± 1.40%). *Quercus* showed the lowest overall intensities, ranging from 19.55 ± 1.50% in April to 45.97 ± 2.99% in July (Fig. 3A). Overall, eight metabolites showed a significant time effect across all species: 7-methylguanine (nucleotide metabolism), nicotinic acid (energy production), 5,7-dihydroxychromone and uric acid (purine derivatives), 3-O-methylgallic acid (stress response), and a C5 sugar alcohol, N-acetylmannosamine, and pyridoxine (osmotic regulation; Fig. 2).

Species had a significant effect on root exudate composition (PERMANOVA: F_2,42_ = 12.74, R^2^ = 0.195, *p* = 0.001), with *Pinus* and *Quercus* forming the most distinct clusters and *Pistacia* positioned in between (Fig. 3B). Time explained the largest proportion of variance (F_3,42_ = 12.08, R^2^ = 0.278, *p* = 0.001), while rain-exclusion treatment also showed a statistically significant but negligible effect with a very small proportion of variance (F_1,42_ = 1.04, R^2^ = 0.008, *p* = 0.001). A significant species × time interaction was detected (F_6,42_ = 2.81, R^2^ = 0.129, *p* = 0.005). Within-species analyses further highlighted the strong role of time, particularly in *Pistacia* (F_3,15_ = 9.56, R^2^ = 0.612, *p* = 0.001), followed by *Pinus* (F_3,16_ = 6.35, R^2^ = 0.495, *p* = 0.001) and *Quercus* (F_3,11_ = 2.346, R^2^ = 0.331, *p* = 0.006). In *Pinus*, samples collected in April clustered together, while July, September and December samples showed higher metabolic overlap. In *Pistacia*, December and April overlapped strongly, while July had a less distinct cluster (Figs. 2 and 3C). See Table S7 for all PERMANOVA results.

### Soil and root bacterial communities

**Fig. 4:**
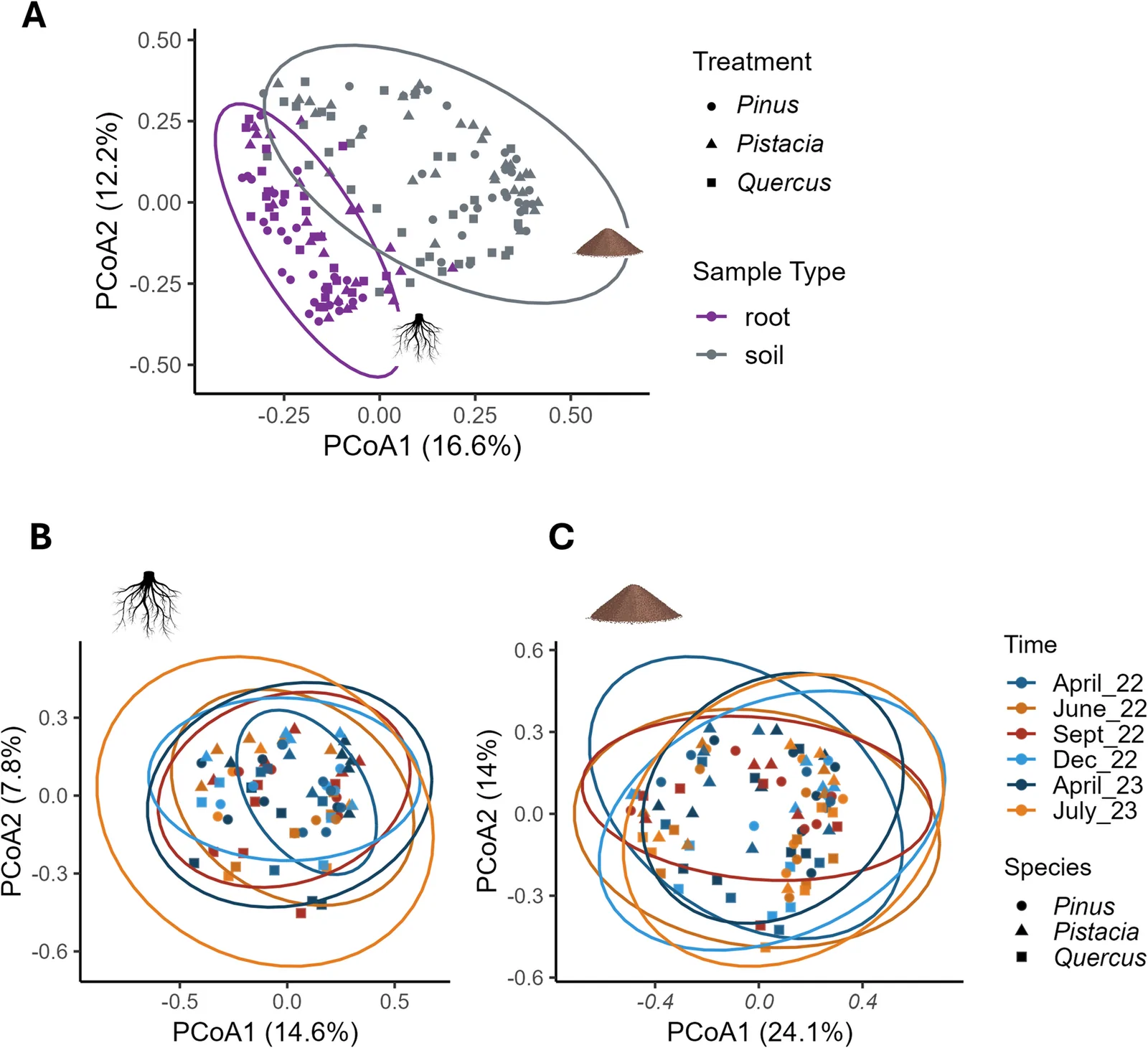
PCoA of root and soil bacterial communities combined (**A**) and separated into root (**B**) and soil (**C**) communities by tree species *Pinus, Pistacia and Quercus* and across seasons (April 22, June 22, Sept 22, Dec 22, April 23, July 23). PCoA was based on Bray–Curtis distances at the ASV level. Ellipses represent 95% confidence intervals

**Fig. 5:**
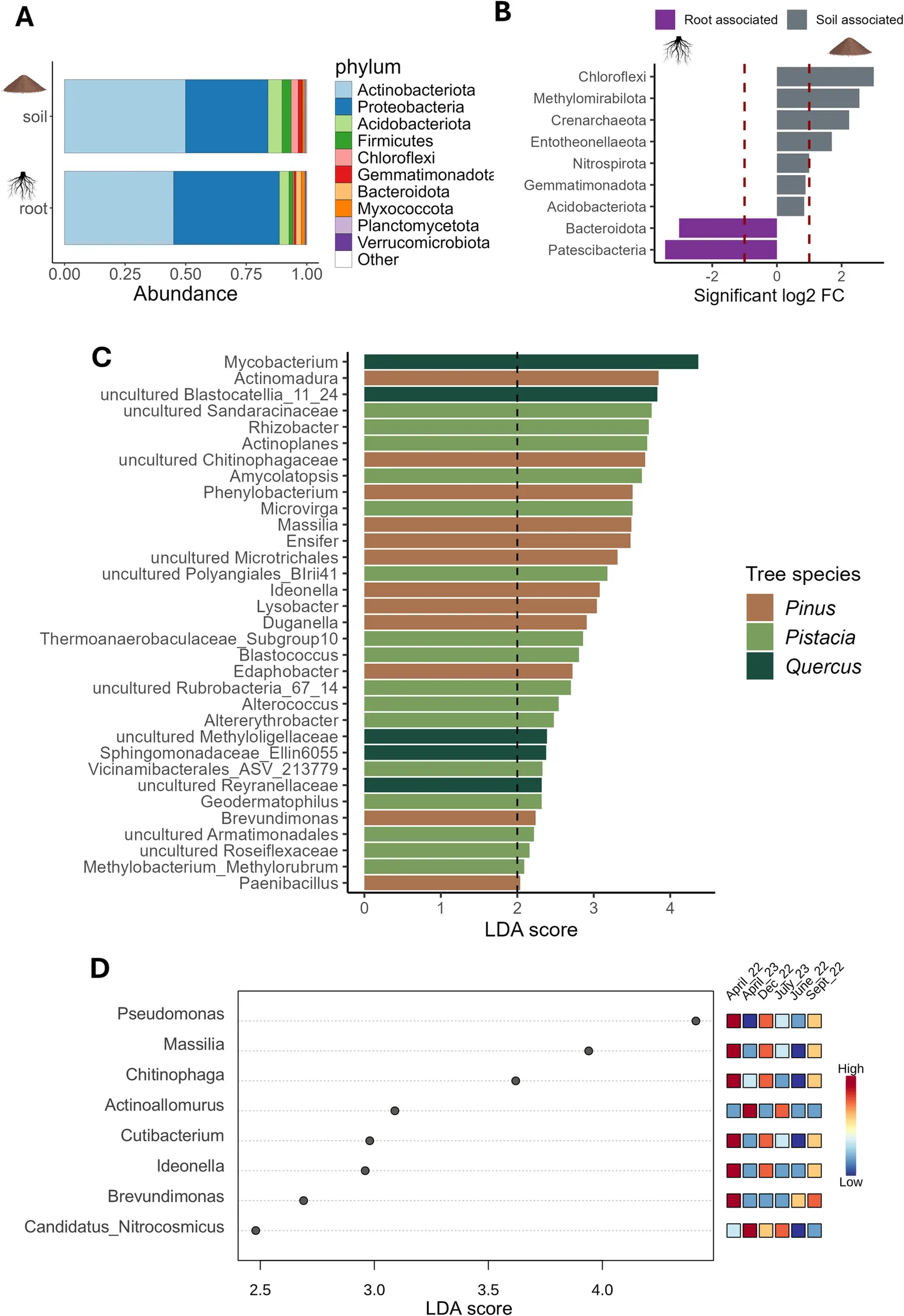
Phylum-level abundances in soil- and root-associated bacterial communities (**A**), differential abundance analysis (MaAsLin2) with log_2_ FC of bacterial phyla enriched in soil (positive values) versus root (negative values; FDR < 0.05; **B**), and LEfSe analysis identifying genera that changed between tree species *Pinus, Pistacia and Quercus* (**C**; color of each bar represents tree specie with the highest LDA score) and across seasons (April 22, June 22, Sept 22, Dec 22, April 23, July 23;** D**) in the root bacterial community (LDA score > 2 and *p*-value < 0.05)

To assess seasonal changes in the belowground community, we measured the soil and root bacterial communities across six time points (April 2022, June 2022, September 2022, December 2022, April 2023, and July 2023) in *Pinus*, *Pistacia*, and *Quercus*. Alpha diversity tended to be lower in the soil bacterial community compared to the rhizosphere (Fig. S3), however this effect was not significant. Similarly, no significant effect was found for tree species, time, and rain-exclusion treatment. Overall, the PCoA showed a clear clustering of the soil and root bacterial communities (PERMANOVA: F_1,101_ = 23.20, R^2^ = 0.117, *p* = 0.001; Fig. 4A), time did not show significant effects (Table S8; Fig. S4A, B). However, tree species had a significant impact on bacterial community composition (F_2,101_ = 4.16, R^2^ = 0.042, *p* = 0.001; Fig. S4C, D); the rain-exclusion treatment also reached significance but accounted for only 0.9% of variation (F_1,101_ = 1.88, R^2^ = 0.009, *p* = 0.020). When performing separate PERMANOVA analyses for the soil and root bacterial communities, tree species remained a significant factor for both subsets, with no significant temporal effects observed, except for a slight difference in root samples from April 2022 (Table S8; Fig. 4B, C). Based on taxonomic classification, *Actinobacteria* and *Proteobacteria* were the dominant phyla, accounting for 45.06 ± 0.14% and 43.6 ± 0.14% in the root bacterial community, and 49.95 ± 0.07% and 34.01 ± 0.09% in the soil bacterial community, respectively (Fig. 5A). Accordingly, the most abundant genera in the root bacterial community were *Streptomyces* (*Actinobacteria*) and *Bradyrhizobium* (*Proteobacteria*), which also showed the highest prevalence within the core root bacterial community (Figs. S5, S6). In the soil, the dominant genus was *Rubrobacter* (*Actinobacteria*), followed by *Bradyrhizobium*. Enrichment analysis revealed increased abundances of *Patescibacteria* and *Bacteroidota* in the root-associated bacterial community, whereas *Chloroflexi*, *Methylomirabilota*, *Entotheonellaeota*, *Nitrospirota*, *Gemmatimonadota* and *Acidobacteriota* were more abundant in the soil-associated bacterial community. *Crenarchaeota* also showed higher abundance in soil (Fig. 5B). LEfSe analysis identified 33 bacterial genera that differed among tree species. Only five genera showed the highest LDA scores for *Quercus* (*Mycobacterium,* uncultured *Blastocatellia_11_24,* uncultured *Methyloligellaceae, Ellin6055* and uncultured *Reyranellaceae;* Fig. 5C), whereas the largest number of genera had their highest LDA scores for *Pistacia*, including *Rhizobacter, Actinoplanes* and *Amycolatopsis.* Across seasons, LEfSe analysis identified eight genera that varied over time; all but two had their highest LDA scores in April 2022, with *Pseudomonas*, *Massilia*, and *Chitinophaga* showing overall the highest LDA scores (Fig. 5D).

### Correlation of metabolites and bacterial communities

**Fig. 6:**
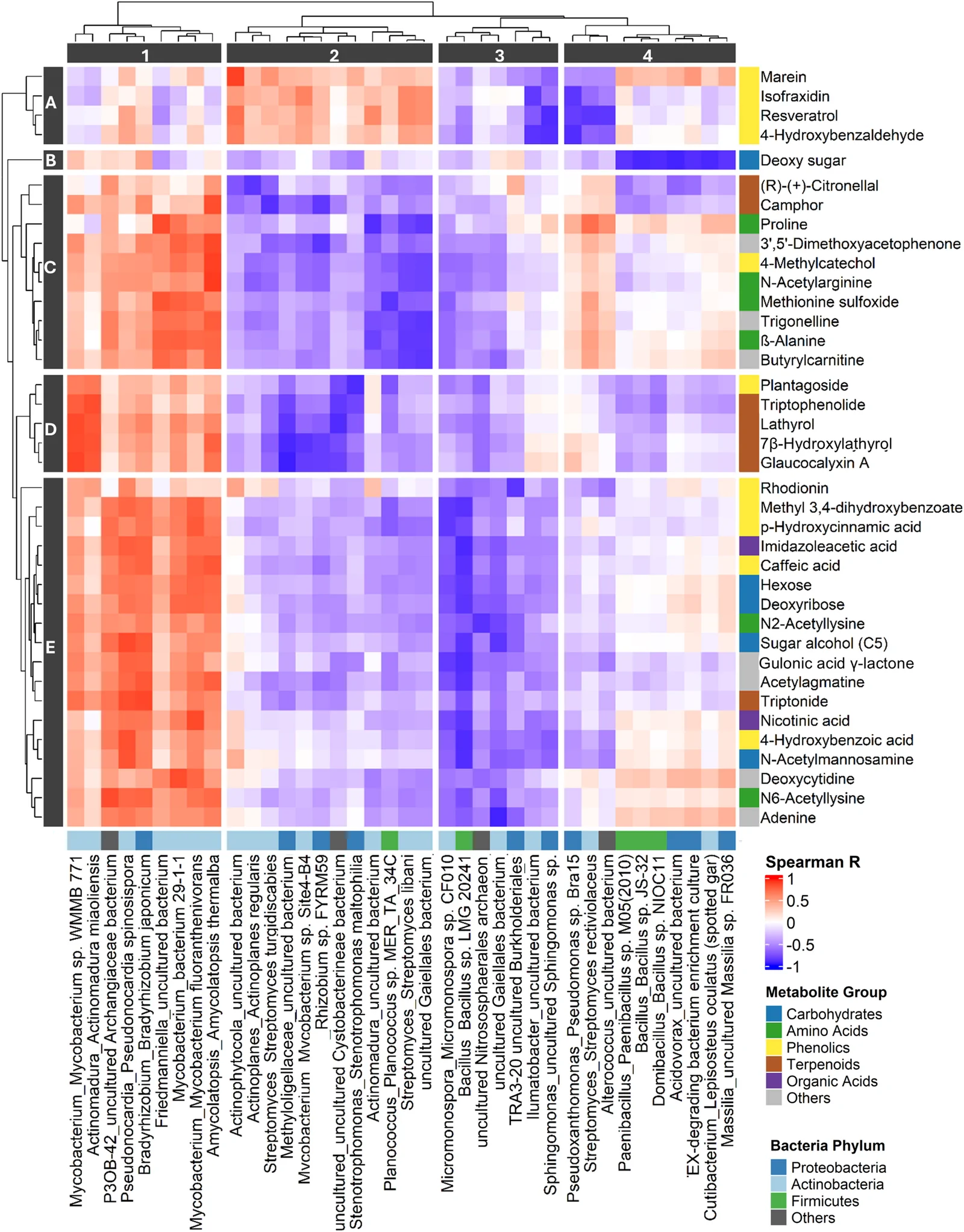
Spearman correlation (R) of rhizosphere bacteria (species-level) and root exudate metabolites. Only bacterial species and root exudate metabolites with at least one strong correlation (|R|> 0.85) were retained. Hierarchical clustering was applied to both rows and columns using Euclidean distance

We assessed associations between drought-responsive metabolites and rhizosphere bacterial communities using Spearman correlations. After filtering for |R|> 0.85, 38 bacterial species and 38 metabolites remained, each showing a strong association with at least one counterpart (Fig. 6). The correlation heatmap displays five metabolite clusters: three small clusters—(A) phenolics (marein, isofraxidin, resveratrol and 4-hydroxybenzaldehyde), (B) only deoxy sugar (C_6_H_12_O_5_) and (D) 1 phenolic (plantagoside) and 4 terpenoids (triptophenolide, lathyrol, 7β-hydroxylathyrol, glaucocalyxin A)—and 2 larger clusters, (C) and (E), comprising 10 and 18 metabolites, respectively. Among the 38 bacterial species, the dominant phyla were 19 *Actinobacteria*, 10 *Proteobacteria*, 5 *Firmicutes*, with 4 other phyla. Bacterial profiles were more coherent than metabolite profiles: most bacterial species tended to be broadly up- or down-correlated across metabolites, whereas metabolite responses were more taxon-specific. Notably, the two large metabolite clusters (C and E) were strongly positively correlated with bacteria cluster 1: *Mycobacterium* sp. WMMB 771, *Actinomadura* miaoliensis, P3OB-42 uncultured *Archangiaceae* bacterium, *Pseudonocardia spinosispora*, *Bradyrhizobium japonicum*, uncultured *Friedmanniella* bacterium, *Mycobacterium* bacterium 29-1-1, *Mycobacterium fluoranthenivorans* and *Amycolatopsis thermalba*. Bacteria clusters 2 and 3 were dominated with negative correlations. Within the correlation matrix, we found two *Pseudomonas* and one *Massilia* species that were correlated with metabolites, consistent with their strong temporal shifts indicated by the highest LDA scores in Fig. 5D. Across metabolite–bacterium pairs we identified more strong negative associations than positive ones (|R|> 0.85). While 10 bacterial species accounted for 15 positive correlations, 28 species contributed to 48 negative correlations, with *Bacillus* sp. LMG 20241 alone accounting for 11 of the negative correlations. Additional taxa with 3–4 strong correlations included *Mycobacterium* sp. WMMB 771 (positive), *Amycolatopsis thermalba* (positive), uncultured *Methyloligellaceae* (negative), and *Gaiellales* (negative). Among metabolites, the deoxy sugar (C_6_H_12_O_5_) exhibited the highest number of strong associations (7 negative), while resveratrol, 4-hydroxybenzaldehyde, proline, lathyrol, 7β-hydroxylathyrol and sugar alcohol (C5) exhibited each 3 associations (positive and negative). Other metabolites showed one or two positive or negative correlations.

**Fig. 7:**
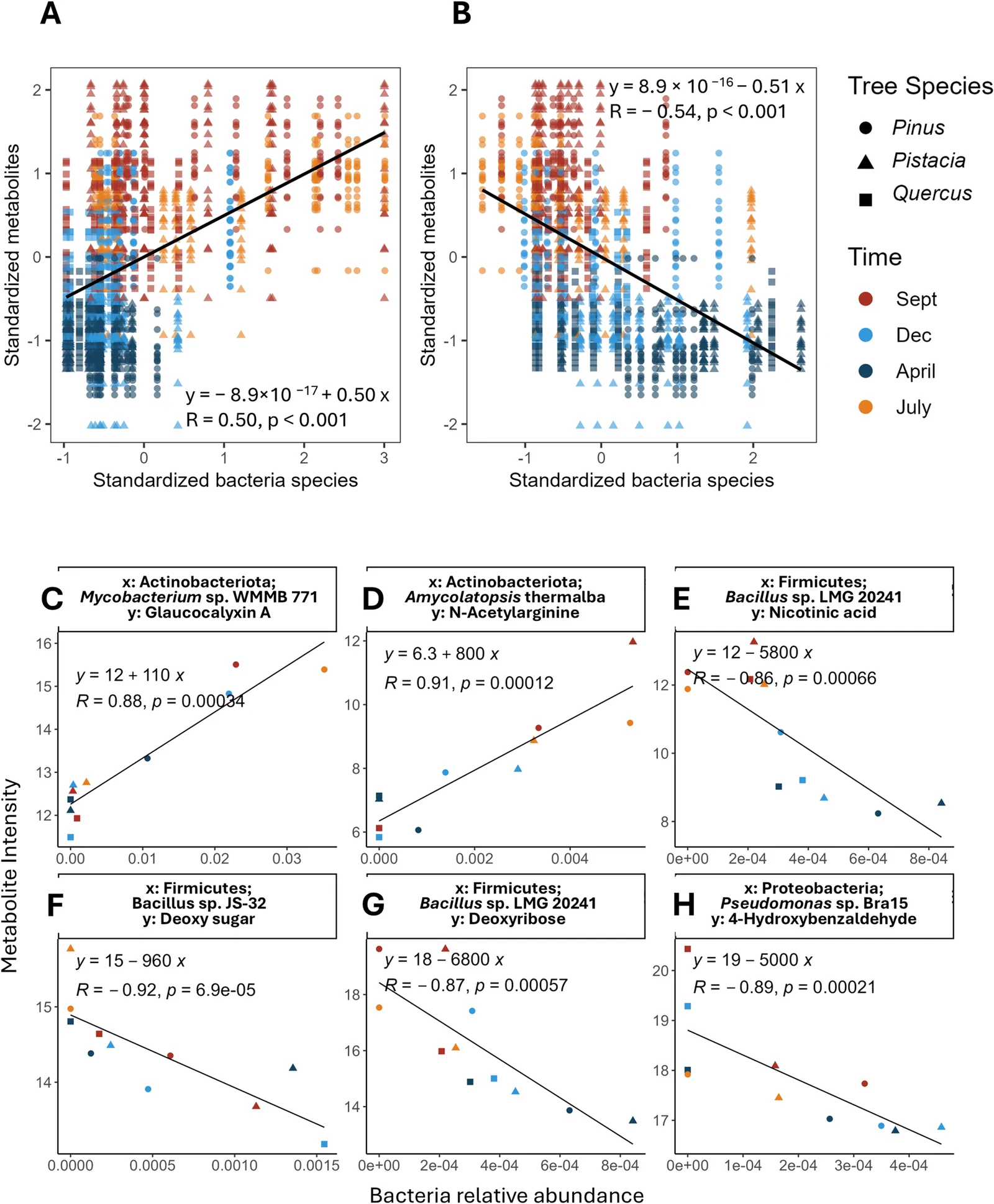
Metabolite–bacteria associations for selected correlations from Fig. 6 across tree species *Pinus, Pistacia and Quercus* and seasons (Sept 22, Dec 22, April 23 and July 23). Panels A and B show subsets of the correlation matrix corresponding to two clusters labelled in Fig. 6: **A** cluster 1E, dominated by positive correlations, and **B** cluster 3E, dominated by negative correlations. Axes represent standardized per-pair z-scores. Panels C–H show example metabolite–bacteria associations. Regression line shows the OLS fit with equation, Spearman’s ρ, and *p*-value

Combining all metabolite–bacteria associations separately for the ‘positive’ cluster 1E and the ‘negative’ cluster 3E (Fig. 7A, B), we found that high metabolite intensities during the dry months strengthened the correlations, although they do not fully drive them. Further, the three terpenoids lathyrol, 7β-hydroxylathyrol and glaucocalyxin A showed a positive association with *Mycobacterium* sp. WMMB 771. *Pinus* contributed significantly to the higher correlation, with both elevated bacterial abundance and metabolite intensity (Figs. 7C, S7). Another positive association was observed between N-acetylarginine and *Amycolatopsis thermalba* (Fig. 7D). Additionally, negative correlations were found, for example, between nicotinic acid and *Bacillus* sp. LMG 20241 (notably, nicotinic acid was among the eight metabolites showing a response to seasonal drought across all three tree species), deoxy sugar and *Bacillus* sp. JS-32, deoxyribose and *Bacillus* sp. LMG 20241, and 4-hydroxybenzaldehyde and *Pseudomonas* sp. Bra15. These are just examples chosen from the larger correlation matrix (Fig. 7E–H).

## Discussion

Our study provides new insights into how root exudation metabolites and rhizosphere bacteria interact in the forest belowground. Seasonal drought led to increased root exudation rates in all three species (*Pinus halepensis*, *Pistacia lentiscus* and *Quercus calliprinos*; Figs. 1C, S1), with species-specific changes in metabolite composition over time (Figs. 2 and 3). Eight metabolites displayed time-dependent responses across all three species (Fig. 2). In contrast, the soil and rhizosphere bacterial communities remained relatively stable, showing no significant seasonal effects (Fig. 4). Despite the relative stability of the bacterial community composition, soil respiration was higher during the wet season compared to the dry season as expected, showing that microbial activity was influenced by seasonal changes (Fig. 1D). Finally, we identified intriguing correlations between specific metabolites and the rhizosphere bacterial community (Figs. 6 and 7).

### Root exudation rate and metabolites across seasons

Dry-season exudation rate exceeded wet-season values in all three tree species, *Pinus*, *Pistacia*, and *Quercus* (Figs. 1C, S1) consistent with our hypothesis and with literature reporting enhanced exudation under water stress [35, 52, 91], although some studies have found down-regulation. As hypothesized, exudate composition in the dry season shifted toward stress-related metabolites (Fig. 2). Higher dry-season metabolite intensities may in part reflect passive leakage from drought-stressed roots, especially because exudation sampling occurred under higher-moisture conditions. However, passive leakage is expected mainly for small primary metabolites, whereas the observed increase of secondary metabolites is more consistent with regulated exudate release than with passive leakage [13]. Importantly, exudation rate and shift in metabolite compositions were reversible: after rewetting (in December and April), rates and most drought-elevated metabolites returned back to baseline (Figs. 1C, 2, 3A). This pattern is typical for moderate drought—secondary metabolites rise transiently, then profiles re-center on primary sugars and organic acids with rewetting—whereas severe or prolonged drought can leave persistent, only partially reversible changes [30].

Of the 89 drought-responsive metabolites, species differed strongly in responsiveness, indicating distinct chemical repertoires for seasonal stress (Figs. 2 and 3). *Pistacia* showed high plasticity, including an ~ 80% decline in metabolite intensity at the onset of the wet season (September to December; Fig. 3A); *Quercus* exhibited the lowest overall intensities and few drought-responsive compounds; *Pinus* was intermediate. These patterns may reflect drought exposure mediated by rooting depth (*Pistacia* < *Pinus* < *Quercus*; [75]), however this gradient was not reflected in leaf water potential or C assimilation in our study area [26, 75]. Consistent with evidence that exudate profiles scale with drought severity (e.g., [81]), percent increase of exudation rate and mean metabolite intensities was highest in shallow-rooted *Pistacia*, least in deep-rooted *Quercus* (Figs. 1C, 3A). Notably, in a controlled drought experiment, *Quercus* did show shifts towards drought-induced secondary metabolites [30]. Thus, rooting depth likely modulates both the magnitude and timing of drought signatures in exudation and exudate metabolites, yielding seasonal, species-specific metabolome reshaping.

Species-specific cluster dominance (e.g., *Quercus* in cluster 1; *Pinus* in cluster 4) supports the view that each tree species has a distinct chemical repertoire with specific effects on belowground interactions. The *Quercus* dominant Cluster 1 (engeletin, isofraxidin, 4-hydroxybenzaldehyde, resveratrol, (E/Z)-polydatin and marein) comprised entirely of phenolic metabolites from the phenylpropanoid pathway consistent with *Quercus*’s phenolic-centric defense [76, 82]. These phenolic exudates may shape rhizosphere microbial communities through both antimicrobial defense activity and signaling roles in plant–microbe interactions [53]; notably, root-secreted coumarins (e.g., isofraxidin) modulate root microbiota, while simple benzenoids such as resveratrol, subsequently converted to 4-hydroxybenzaldehyde, function both as allelochemicals and as microbial substrates, known as plant drought adaptation strategy [70], [16, 32, 45, 86]. Notably, both benzenoids were drought-responsive only in *Quercus*, indicating a *Quercus*-specific drought response. Cluster 4 included multiple diterpenoids (6 out of 9 metabolites), indicating a strong representation of resin-type conifer defenses that typically increase during stress or wounding; this pattern supports a shift toward defensive allocation in the dry season and aligns with the known defense chemistry of pines [43, 57].

Across all three species, eight metabolites were consistently drought responsive: 7-methylguanine, nicotinic acid, 5,7-dihydroxychromone, uric acid, 3-O-methylgallic acid, a sugar alcohol (polyol), N-acetylmannosamine, and pyridoxine, indicating a conserved seasonal adjustment in Mediterranean trees. Several of these compounds have established roles in plant stress responses and plant–microbe signaling. Notably, the two vitamins, nicotinic acid and pyridoxine, are crucial cofactors in plant metabolism and stress responses, particularly in maintaining cellular redox balance during drought stress [21, 25, 41]. Specifically, exogenously applied nicotinic acid enhanced antioxidant defense mechanisms and alleviated drought-induced stress in plants [3, 41]. Their consistent seasonal variation may therefore indicate metabolic adjustment to water availability across diverse Mediterranean taxa. The phenolics 5,7-dihydroxychromone and methylgallic acid are consistent with secondary-metabolite–based protection and antimicrobial activity, as 5,7-dihydroxychromone has reported antifungal activity against soil-pathogenic fungi, while methylgallic acid derivatives show antioxidant and antimicrobial activities [1, 83]. Collectively, these shared drought-responsive metabolites suggest a convergent chemical adjustment during dry periods, potentially linked to redox regulation and defense-related rhizosphere interactions, although these mechanisms remain to be experimentally tested in our system.

### Decoupling of root exudates and rhizosphere bacterial community

Contrary to our second hypothesis, the soil and rhizosphere bacterial communities of *Pinus*, *Pistacia*, and *Quercus* remained stable at the taxonomic level despite pronounced seasonal shifts in root exudates (Fig. 4B, C). Although drought is frequently associated with major restructuring of the rhizosphere microbiome, often increasing relative abundance of drought-tolerant groups (e.g. *Actinobacteria* and *Bacilli*) while reducing groups like *Acidobacteria* and *Bacteroidota* [56], both soil and root bacterial communities in our Mediterranean forest system were comparatively resistant to seasonal drought. This contrasts with many crop and temperate systems but is consistent with long-term adaptation to predictable summer drought in Mediterranean forests [56]. These observations are further supported by Anthony et al. [4], who showed that mean annual precipitation is tightly linked to microbial composition. First, plants maintain and even increase exudation under drought (Fig. 1C), buffering bacterial communities from moisture limitation and possibly facilitating rapid post-drought recovery—mechanistically decoupling plant C fluxes from immediate bacterial turnover. This has been shown in a grassland study, where bacterial activity decreased during drought despite continued plant C release, yet this maintained root exudation enabled rapid post-drought recovery upon rewetting [39]. Second, ecosystem context matters: in systems with relatively stable edaphic properties (e.g., some tropical dry forests), microbial composition can be seasonally steady even as abiotic conditions oscillate [77]; in more extreme deserts, endosphere communities track climatic pulses more tightly [96]. Our results align with the former pattern, resistance in composition despite hydrologic swings, highlighting context dependence in plant–bacteria coupling. Despite overall compositional stability, host tree explained ~ 4% of bacterial community variation (Table S8). Consistent with the exudate patterns, *Quercus* showed the fewest genera that distinguished its root bacterial community from the other tree species in the LEfSe analysis (5 out of 33; Fig. 5C), suggesting the more conservative or less distinctive root metabolome (Fig. 2) possibly overlaps with a less distinctive root bacterial community. Thus, even without seasonal drought-driven bacterial community restructuring, tree species maintained distinct bacterial assemblages. In sum, Mediterranean trees host stable yet species-specific rhizosphere consortia that appear resilient to seasonal drought. Functionally, this stability may mask underlying activity shifts. Soil respiration, for example, was higher in the wet compared to the dry season, confirming bacterial sensitivity to soil moisture (Fig. 1D), typical for Mediterranean climates where water pulses dominate soil respiration dynamics [74]. Together, the evidence supports taxonomic resistance with functional adjustment under seasonal drought, plausibly aided by plant C allocation strategies that stabilize microbe partners through dry periods. Climate-change legacies suggest that *Proteobacteria* underpin the resilience of soil multifunctionality after drought, consistent with functional, rather than compositional, responses in our sites [19]. In that study, overall community composition and richness changed little, reinforcing the view that drought legacies act chiefly through shifts in function/activity rather than taxonomic turnover.

### Correlation between root exudates and bacterial communities

Our third hypothesis of metabolite-specific bacteria recruitment was partially supported. One-to-one attractants were rare, but many bacterial species showed broad positive or negative correlations to most metabolites, which can be interpreted as mixture-level chemical filtering rather than single-compound effects (Fig. 6). While a few strong metabolite–bacteria associations suggest strain-specific niches, the dominant signal was response to the overall exudate cocktail, as expected given that roots release complex mixtures and successful colonists are those able to tolerate i.e. phenolic loads and co-metabolize diverse substrates. Interestingly, most metabolite– bacteria associations were negative (48 out of 63; |R|> 0.85; Fig. 6), which can be interpreted that chemical filtering outweighed targeted recruitment (but see alternative interpretations in Sect. *Limitations and future perspectives*). Among all metabolites, deoxy sugar (C_6_H_12_O_5_) showed the most negative associations with bacteria (Fig. 6). Although deoxy sugars can serve as labile C, certain deoxy-hexose analogs act as antimetabolites and inhibit bacterial growth, e.g., of pathogenic bacteria (for example *Escherichia coli* and *Bacillus anthracis*; [22, 79, 85]). Our study shows similar inhibition, particularly in *Acidovorax* and *Pseudomonas*, both known to contain plant pathogenic species [36, 80]. However, deoxy sugars may not only suppress potential pathogens but also beneficial bacteria, as many of the bacterial genera inhibited by deoxy sugar in our study are not typically considered plant pathogens.

Further, significant negative correlations were observed between the *Bacillus* sp. LMG 20241 (phylum *Firmicutes*, order *Bacillales*) and the metabolite nicotinic acid and deoxyribose (Fig. 7E, G). This relationship suggests that *Bacillus* abundance decreases when nicotinic acid levels are elevated, possibly reflecting competition for NAD⁺ precursors or inhibition by high vitamin B₃ concentrations. Such interactions are consistent with reports of *Bacillus* involvement in vitamin metabolism and redox balance in the rhizosphere. Additionally, previous experiments indicated that nicotine (a product of nicotinic acid) does not promote the growth of *Bacillus* in laboratory settings [49, 59, 95]. Similarly, excess deoxyribose has been shown to be toxic to certain bacterial strains by inhibiting key radical enzymes, disrupting critical metabolic processes. For example, *Bacillus thuringiensis* can salvage 5-deoxyribose byproducts, but mutants lacking this salvage pathway exhibit growth inhibition in the presence of deoxyribose [7]. This aligns with our own observations, where the negative correlation between *Bacillus* sp. LMG 20241 and deoxyribose suggests that deoxyribose accumulation could similarly interfere with *Bacillus* growth. Further, we found negative correlations for 4-hydroxybenzaldehyde with ***Pseudomonas*** **sp. Bra15**. Although many ***Pseudomonas*** **spp**. catabolize lignin-related phenolics, including 4-hydroxybenzaldehyde/4-hydroxybenzoate, high concentrations can be inhibitory, implying dose-dependent outcomes at the metabolite–microbe interface [18].

We found that three *Mycobacterium* strains positively correlated with metabolites, such as *Mycobacterium* sp. WMMB 771 with three terpenoids (glaucocalyxin A, lathyrol and 7β-hydroxylathyrol) which are known for their antimicrobial properties and involvement in plant defense from herbivores and pathogens. Under environmental stress, some *Mycobacterium* strains may play beneficial roles, aligning with previous reports of *Mycobacterium* species promoting plant growth in other systems, such as coffee and tomato [10, 20]. Interestingly, *Mycobacterium* species were the most enriched in the root bacterial community of *Quercus* (Fig. 5C), supporting their ability to survive in harsh environmental conditions [61]. Notably, however, the specific positive association between *Mycobacterium* sp. WMMB 771 and the three terpenoids was primarily driven by pine trees (Fig. S7). In addition, we also found a negative correlation with another *Mycobacterium* species, underlining that strain identity trumps genus stereotypes [63]. While *Mycobacterium* species showed increased abundance under global change factors such as drought and salinity, many of these species also contained virulence factor genes, indicating a potential for pathogenicity [64]. This highlights the dual nature of *Mycobacterium*, where some strains may act as pathogens, while others can play beneficial roles in plant health.

Finally, phenolic compounds, such as caffeic and benzoic acid, identified in our study, are key exudate metabolites that restructure bacterial communities and enhance soil organic matter turnover, particularly when labile C (e.g., glucose) is available [97]. These phenolics can drive bacterial reconfiguration, with negative bacterial correlations dominating in our study, which might reflect the inhibitory effects of high phenolic and aromatic aldehyde levels. For example, resveratrol induces membrane damage and disrupts biofilm formation [84] and was correlated with inhibition of three bacterial species in our study, whereas context-specific positive correlation also occurred.

### Limitations and future perspectives

First, our bacterial data are amplicon-based, providing taxonomic resolution but limited functional insight. Omics (metatranscriptomics/proteomics), stable-isotope probing, or substrate-responsive assays are needed to detect activity shifts that do not manifest as compositional change. Second, exudate collections may not perfectly represent in-soil concentrations given rapid sorption/turnover; pairing exudate and soil metabolomics would strengthen causal inference. In addition, metabolites detected in exudates may be partially microbial in origin or rapidly transformed after release. Third, correlation structure was strongly season-driven; while we highlighted examples robust to this, causality requires targeted experiments (e.g., adding individual metabolites to microcosms or gnotobiotic systems) to separate co-fluctuation from interaction. Fourth, correlations could stem from alternative scenarios, e.g., negative correlations could also reflect microbial uptake of metabolites, and higher bacterial abundance may lead to faster depletion of certain compounds. Overall, our results indicate that drought-induced shifts in root exudate composition act as finely tuned metabolic cues that selectively reshape bacterial interactions. Obviously, more work is needed to decipher specific metabolite-bacteria interactions. Future work will examine the effect of ^13^C-labelled artificial root exudates on bacterial composition and biomass in the forest soil, incubated in tubes under controlled conditions.

## Supplementary Information

Additional file1 (PDF 1737 KB)

## Data Availability

All data is available in an open repository https://doi.org/10.5281/zenodo.17662263 (https://zenodo.org/records/17662264). DNA sequence data is available in NCBI repository (BioProject accession: PRJNA1373004 https://dataview.ncbi.nlm.nih.gov/object/PRJNA1373004?reviewer=ssg0b6sontfcbl11skrb38hkia). The metabolomics data reported in this paper have been deposited in the OMIX, China National Center for Bioinformation / Beijing Institute of Genomics, Chinese Academy of Sciences (https://ngdc.cncb.ac.cn/omix: accession no. OMIX014435). No special code or custom scripts were used in this study.
