# Supplementary material for: Pine root exudate terpenoids associate with Mycobacterium sp. under summer drought in a mixed Mediterranean forest
Source: Environ Microbiome. 2026 Aug 3;21:134. doi: 10.1186/s40793-026-00921-8 (PMC13757234; doi:10.1186/s40793-026-00921-8)
Supplement: Supplementary file 1 [file 40793_2026_921_MOESM1_ESM.pdf]

Supplementary Tables

Table S1. Stem diameter (cm) and height (m) of trees of control and rain exclusion (RE) plots (mean ± SD; n=3).

		**Stem diameter (cm)**	**Height (m)**
Pinus	control	39.47 ± 3.46	14.07 ± 1.51
	RE	37.84 ± 10.84	13.13 ± 2.29
Pistacia	control	2.57 ± 0.81	1.74 ± 0.31
	RE	2.74 ± 0.99	1.82 ± 0.40
Quercus	control	11.44 ± 4.56	4.03 ± 0.33
	RE	13.03 ± 5.14	4.78 ± 1.59

Table S2. Number of replicates for root exudation rate, metabolite profile of root exudates, soil respiration, root and soil microbiomes for *Pinus* (Ph), *Pistacia* (Pl) and *Quercus* (Qc).

Measurement	Time	Species	Control plot (n)	Rain-excl. plot (n)	Total (n)
**Root exudation rate** (n_{total} = 3-6)	July_21	Ph	3	2	5
		Pl	3	2	5
		Qc	1	3	4
	Aug_21	Ph	2	3	5
		Pl	1	2	3
		Qc	2	2	4
	Sept_21	Ph	3	3	6
		Pl	2	2	4
		Qc	3	2	5
	Oct_21	Ph	3	3	6
		Pl	2	2	4
		Qc	2	3	5
	Nov_21	Ph	3	3	6
		Pl	2	1	3
		Qc	3	2	5
	Dec_21	Ph	3	3	6
		Pl	3	1	4
	Jan_22	Ph	3	3	6
		Pl	3	1	4
		Qc	3	1	4
	Feb_22	Ph	3	3	6
		Qc	2	2	4
	March_22	Ph	3	3	6
	April_22	Ph	3	3	6
		Qc	3	2	5
	May_22	Ph	3	3	6
	July_22	Ph	3	2	5
	June_22	Ph	3	3	6
		Qc	3	2	5
	Sept_22	Ph	3	3	6
		Pl	3	2	5
		Qc	3	3	6
	Dec_22	Ph	3	3	6
		Pl	3	3	6
		Qc	3	2	5
	April_23	Ph	3	3	6
		Pl	3	3	6
		Qc	3	1	4
	July_23	Ph	3	3	6
		Pl	3	3	6
		Qc	3	1	4

Measurement	Time	Species	Control plot (n)	Rain-excl. plot (n)	Total (n)
**Metabolite analysis** (n_{total} = 4-6)	Sept_22	Ph	3	3	6
		Pl	3	2	5
		Qc	3	3	6
	Dec_22	Ph	3	3	6
		Pl	3	3	6
		Qc	3	2	5
	April_23	Ph	3	3	6
		Pl	3	3	6
		Qc	3	1	4
	July_23	Ph	3	3	6
		Pl	3	3	6
		Qc	3	1	4
**Soil Respiration** (n_{total} = 4)	Sept_22	Ph	2	2	4
		Pl	2	2	4
		Qc	2	2	4
	April_23	Ph	2	2	4
		Pl	2	2	4
		Qc	2	2	4
**Microbiome root** (n_{total} = 2-6)	April_22	Ph	3	3	6
		Pl	0	2	2
		Qc	2	2	4
	June_22	Ph	0	3	3
		Pl	2	3	5
		Qc	3	1	4
	Sept_22	Ph	1	3	4
		Pl	3	2	5
		Qc	3	3	6
	Dec_22	Ph	3	3	6
		Pl	3	2	5
		Qc	3	2	5
	April_23	Ph	2	3	5
		Pl	3	3	6
		Qc	3	2	5
	July_23	Ph	0	2	2
		Pl	1	1	2
		Qc	0	2	2
**Microbiome soil** (n_{total} = 3-6)	April_22	Ph	3	2	5
		Pl	3	3	6
		Qc	3	2	5
	June_22	Ph	3	1	4
		Pl	3	2	5
		Qc	2	3	5
	Sept_22	Ph	3	3	6
		Pl	2	2	4
		Qc	3	2	5
	Dec_22	Ph	1	2	3
		Pl	3	2	5
		Qc	3	3	6
	April_23	Ph	3	3	6
		Pl	3	2	5
		Qc	3	3	6
	July_23	Ph	2	3	5
		Pl	3	2	5
		Qc	3	3	6

Table S3. Summary of measurement campaigns conducted between July 2021 and July 2023, including root exudation rates, metabolite profiles of root exudates, soil and root microbiomes, and soil respiration, for *Pinus* (Ph), *Pistacia* (Pl) and *Quercus* (Qc).

Month	Root exudation rate	Root exudation metabolites	Soil and Root Microbiome	Soil Respiration
**July 2021**	Ph, Pl, Qc			
**Aug 2021**	Ph, Pl, Qc			
**Sept 2021**	Ph, Pl, Qc			
**Oct 2021**	Ph, Pl, Qc			
**Nov 2021**	Ph, Pl, Qc			
**Dec 2021**	Ph, Pl, Qc			
**Jan 2022**	Ph, Pl, Qc			
**Feb 2022**	Ph, Qc			
**Mar 2022**	Ph			
**April 2022**	Ph, Qc		Ph, Pl, Qc	
**May 2022**	Ph			
**June 2022**	Ph, Qc		Ph, Pl, Qc	
**Sept 2022**	Ph, Pl, Qc	Ph, Pl, Qc	Ph, Pl, Qc	Ph, Pl, Qc
**Dec 2022**	Ph, Pl, Qc	Ph, Pl, Qc	Ph, Pl, Qc	
**April 2023**	Ph, Pl, Qc	Ph, Pl, Qc	Ph, Pl, Qc	Ph, Pl, Qc
**July 2023**	Ph, Pl, Qc	Ph, Pl, Qc	Ph, Pl, Qc	

Table S4. Soil chemistry of pH and mineral content (Cl, Mg, N-NO_3, N-NH_4, Olsen-P concentration) of control and rain exclusion (RE) plots (mean ± SD; n=2-3).

Tree specie	Treatment	pH	Cl (mg kg^{-1})	Mg (mg kg^{-1})	N-NO_3 (mg kg^{-1})	N-NH_4 (mg kg^{-1})	Olsen-P (mg kg^{-1})
	control	7.91 ± 0.04	41.32 ± 22.67	19.13 ± 1.1	7.71 ± 8.5	0.74 ± 0.58	8.07 ± 6.42
Pinus	RE	7.61 ± 0.35	77.83 ± 18.74	31.33 ± 0.12	1.4 ± 1.6	1.07 ± 0.46	2.23 ± 2.39
	control	8.04 ± 0.2	75.79 ± 43.98	8.59 ± 7.39	8.46 ± 5.43	0.34 ± 0.26	8.79 ± 4.98
Pistacia	RE	7.85 ± 0.1	44.04 ± 18.3	13.84 ± 2.86	3.45 ± 1.13	0.56 ± 0.35	7.65 ± 6.84
	control	7.86 ± 0.03	63.63 ± 12.4	20.57 ± 18.52	6.73 ± 10.41	2.17 ± 1.4	6.76 ± 10.7
Quercus	RE	8.12 ± 0.05	28.54 ± 16.61	8.22 ± 8.95	1.47 ± 1.14	0.6 ± 0.01	3.58 ± 4.6

Table S5. Results of decrease of unassigned reads after database enrichment of microbiome analysis.

samples	unassgined reads before enrichment	unassigned reads after enrichment	Absolute_change	Percent_change
R_100A99_TK_soil_131124_S65	0.15	0.14	-0.01	-9.89
R_103D99_TK_soil_131124_S68	0.32	0.30	-0.02	-5.92
R_105F99_TK_soil_131124_S70	0.31	0.29	-0.03	-8.76
R_106G99_TK_soil_131124_S71	0.34	0.26	-0.07	-21.97
R_10C710_TK_soil_131124_S241	0.21	0.19	-0.02	-8.64
R_112E109_TK_soil_131124_S77	0.18	0.16	-0.02	-10.43
R_113F109_TK_soil_131124_S78	0.21	0.18	-0.03	-14.83
R_114G109_TK_soil_131124_S79	0.17	0.15	-0.02	-11.24
R_115H109_TK_soil_131124_S80	0.26	0.23	-0.03	-10.09
R_116A119_TK_soil_131124_S81	0.25	0.23	-0.02	-9.01
R_117B119_TK_soil_131124_S82	0.16	0.14	-0.02	-10.81
R_121F119_TK_soil_131124_S86	0.14	0.13	-0.01	-8.97
R_122G119_TK_soil_131124_S87	0.24	0.23	-0.02	-7.79
R_123H119_TK_soil_131124_S88	0.28	0.26	-0.02	-7.25
R_124A110_TK_soil_131124_S199	0.19	0.17	-0.02	-9.98
R_125B110_TK_soil_131124_S200	0.20	0.18	-0.02	-9.49
R_127D110_TK_soil_131124_S202	0.39	0.37	-0.02	-4.73
R_129F110_TK_soil_131124_S204	0.17	0.15	-0.02	-12.54
R_12D710_TK_soil_131124_S242	0.19	0.16	-0.02	-12.17
R_130G110_TK_soil_131124_S205	0.12	0.11	-0.01	-10.24
R_131H110_TK_soil_131124_S206	0.14	0.13	-0.01	-10.34
R_132A210_TK_soil_131124_S207	0.18	0.16	-0.02	-10.76
R_133B210_TK_soil_131124_S208	0.27	0.25	-0.02	-8.80
R_134C210_TK_soil_131124_S209	0.21	0.18	-0.02	-11.48
R_135D210_TK_soil_131124_S210	0.26	0.23	-0.03	-12.10
R_138G210_TK_soil_131124_S213	0.32	0.30	-0.03	-8.21
R_139H210_TK_soil_131124_S214	0.15	0.14	-0.01	-9.79
R_141B310_TK_soil_131124_S216	0.20	0.18	-0.02	-9.90
R_143D310_TK_soil_131124_S218	0.26	0.25	-0.01	-5.04
R_144E310_TK_soil_131124_S219	0.13	0.12	-0.02	-11.88
R_146F310_TK_soil_131124_S220	0.25	0.23	-0.02	-7.74
R_149H310_TK_soil_131124_S222	0.17	0.16	-0.01	-6.32
R_150A410_TK_soil_131124_S223	0.24	0.22	-0.02	-8.88
R_151B410_TK_soil_131124_S224	0.16	0.14	-0.02	-9.88
R_155D410_TK_soil_131124_S226	0.26	0.25	-0.01	-3.58
R_156E410_TK_soil_131124_S227	0.24	0.20	-0.04	-18.31
R_157F410_TK_soil_131124_S228	0.14	0.12	-0.02	-13.75
R_158G410_TK_soil_131124_S229	0.17	0.15	-0.02	-12.28
R_162H410_TK_soil_131124_S230	0.15	0.14	-0.01	-8.60

samples	unassgined reads before enrichment	unassigned reads after enrichment	Absolute_change	Percent_change
R_178A610_TK_soil_131124_S231	0.14	0.12	-0.02	-14.79
R_188B610_TK_soil_131124_S232	0.29	0.28	-0.01	-2.12
R_189C610_TK_soil_131124_S233	0.18	0.17	-0.02	-8.54
R_18B19_TK_soil_131124_S2	0.46	0.34	-0.12	-25.75
R_190D610_TK_soil_131124_S234	0.13	0.12	-0.02	-11.70
R_203A910_TK_root_131124_S247	0.34	0.28	-0.06	-18.02
R_204B910_TK_root_131124_S248	0.29	0.25	-0.04	-12.91
R_205C910_TK_root_131124_S249	0.27	0.23	-0.04	-13.43
R_206D910_TK_root_131124_S250	0.20	0.17	-0.03	-12.97
R_215E1010_TK_root_131124_S259	0.31	0.21	-0.10	-31.21
R_216F1010_TK_root_131124_S260	0.23	0.18	-0.05	-20.07
R_226F1110_TK_root_131124_S268	0.18	0.13	-0.05	-27.48
R_227G1110_TK_root_131124_S269	0.25	0.20	-0.05	-18.43
R_230A111_TK_root_131124_V1_S89	0.14	0.10	-0.03	-24.50
R_231B111_TK_root_131124_S91	0.24	0.17	-0.07	-29.77
R_232C111_TK_root_131124_V2_S93	0.19	0.16	-0.03	-15.29
R_235F111_TK_root_131124_S97	0.18	0.14	-0.04	-23.90
R_236G111_TK_root_131124_S98	0.27	0.16	-0.11	-40.08
R_237H111_TK_root_131124_S99	0.21	0.18	-0.04	-17.24
R_238A211_TK_root_131124_S100	0.36	0.25	-0.11	-30.00
R_240C211_TK_root_131124_S102	0.22	0.12	-0.10	-47.31
R_241D211_TK_root_131124_S103	0.13	0.12	-0.01	-5.27
R_246G211_TK_root_131124_V2_S107	0.22	0.20	-0.02	-7.13
R_247H211_TK_root_131124_S108	0.22	0.19	-0.03	-14.69
R_248A311_TK_root_131124_S109	0.21	0.20	-0.02	-7.14
R_252C311_TK_root_131124_S111	0.17	0.16	-0.01	-3.48
R_253D311_TK_root_131124_S112	0.23	0.19	-0.04	-16.70
R_254E311_TK_root_131124_S113	0.18	0.16	-0.02	-9.78
R_256G311_TK_root_131124_S115	0.43	0.30	-0.13	-31.06
R_258A411_TK_root_131124_S117	0.17	0.15	-0.02	-8.94
R_25G19_TK_soil_131124_S7	0.14	0.12	-0.01	-10.11
R_260B411_TK_root_131124_S118	0.35	0.24	-0.12	-32.62
R_262C411_TK_root_131124_S119	0.24	0.22	-0.02	-8.05
R_263D411_TK_root_131124_S120	0.14	0.14	0.00	-2.41
R_264E411_TK_root_131124_S121	0.44	0.33	-0.11	-24.17
R_265F411_TK_root_131124_S122	0.21	0.19	-0.02	-10.43
R_266G411_TK_root_131124_S123	0.21	0.20	-0.02	-7.32
R_267H411_TK_root_131124_S124	0.32	0.25	-0.07	-21.08
R_26H19_TK_soil_131124_S8	0.16	0.13	-0.03	-19.06
R_271B511_TK_root_131124_S126	0.30	0.27	-0.04	-11.51
R_272C511_TK_root_131124_S127	0.35	0.30	-0.05	-15.52
R_273D511_TK_root_131124_S128	0.21	0.18	-0.03	-13.42
R_274E511_TK_root_131124_S129	0.17	0.14	-0.03	-17.04
R_275F511_TK_root_131124_S130	0.28	0.24	-0.05	-16.08
R_278A611_TK_root_131124_S133	0.16	0.14	-0.02	-13.13
R_280C611_TK_root_131124_S135	0.23	0.23	0.00	-2.04
R_281D611_TK_root_131124_S136	0.30	0.29	-0.02	-5.84
R_283F611_TK_root_131124_S138	0.18	0.17	-0.01	-7.07
R_284G611_TK_root_131124_S139	0.07	0.07	-0.01	-7.40
R_287B711_TK_root_131124_S142	0.29	0.22	-0.07	-23.66
R_288C711_TK_root_131124_S143	0.29	0.22	-0.07	-24.25
R_289D711_TK_root_131124_S144	0.34	0.23	-0.11	-32.12
R_291E711_TK_root_131124_S145	0.19	0.15	-0.04	-20.28
R_295A811_TK_root_131124_S149	0.17	0.15	-0.02	-11.26
R_296B811_TK_root_131124_S150	0.19	0.15	-0.04	-22.19
R_298D811_TK_root_131124_S152	0.26	0.17	-0.09	-35.99
R_299E811_TK_root_131124_S153	0.14	0.12	-0.01	-10.79
R_300F811_TK_root_131124_S154	0.33	0.24	-0.09	-26.60
R_301G811_TK_root_131124_S155	0.13	0.12	-0.01	-5.49
R_303A911_TK_root_131124_S157	0.27	0.17	-0.10	-36.20
R_304B911_TK_root_131124_S158	0.15	0.14	-0.01	-7.93
R_307E911_TK_root_131124_S161	0.21	0.15	-0.06	-26.90
R_308F911_TK_root_131124_S162	0.15	0.13	-0.01	-9.13
R_315C1011_TK_root_131124_S167	0.21	0.17	-0.04	-18.32
R_316D1011_TK_root_131124_S168	0.17	0.15	-0.02	-10.76
R_317E1011_TK_root_131124_S169	0.34	0.30	-0.04	-11.80
R_318F1011_TK_root_131124_S170	0.17	0.14	-0.03	-19.16
R_319G1011_TK_root_131124_S171	0.28	0.25	-0.04	-13.42
R_320H1011_TK_root_131124_S172	0.11	0.10	-0.01	-6.14
R_325E1111_TK_root_131124_S177	0.10	0.10	0.00	-4.23
R_326F1111_TK_root_131124_S178	0.12	0.12	0.00	-0.45
R_328G1111_TK_root_131124_S179	0.11	0.11	0.00	-1.32

samples	unassgined reads before enrichment	unassigned reads after enrichment	Absolute_change	Percent_change
R_329H1111_TK_root_131124_S180	0.09	0.08	-0.01	-9.38
R_32D29_TK_soil_131124_S12	0.18	0.16	-0.02	-12.39
R_330A1210_TK_root_131124_S271	0.08	0.06	-0.03	-32.17
R_332B1210_TK_root_131124_S272	0.07	0.03	-0.03	-51.00
R_380C1210_TK_root_131124_S273	0.29	0.24	-0.05	-17.98
R_384E1210_TK_root_131124_S275	0.18	0.16	-0.03	-15.35
R_43B39_TK_soil_131124_S18	0.17	0.16	-0.01	-8.29
R_44C39_TK_soil_131124_S19	0.27	0.25	-0.02	-7.69
R_45D39_TK_soil_131124_S20	0.18	0.16	-0.02	-9.08
R_47E39_TK_soil_131124_S21	0.30	0.29	-0.01	-3.39
R_48F39_TK_soil_131124_S22	0.16	0.14	-0.02	-12.31
R_49G39_TK_soil_131124_S23	0.18	0.16	-0.02	-10.36
R_50H39_TK_soil_131124_S24	0.24	0.21	-0.03	-10.62
R_52B49_TK_soil_131124_S26	0.27	0.25	-0.02	-7.54
R_53C49_TK_soil_131124_S27	0.33	0.31	-0.02	-6.30
R_55E49_TK_soil_131124_S29	0.16	0.14	-0.02	-11.16
R_57F49_TK_soil_131124_S30	0.17	0.16	-0.02	-9.90
R_58G49_TK_soil_131124_S31	0.17	0.15	-0.01	-8.82
R_59H49_TK_soil_131124_S32	0.33	0.31	-0.01	-4.01
R_5G610_TK_soil_131124_S237	0.15	0.14	-0.01	-8.01
R_60A59_TK_soil_131124_S33	0.28	0.26	-0.02	-6.99
R_61B59_TK_soil_131124_S34	0.18	0.15	-0.03	-17.98
R_63D59_TK_soil_131124_S36	0.21	0.20	-0.02	-8.20
R_64E59_TK_soil_131124_S37	0.23	0.21	-0.02	-9.27
R_65F59_TK_soil_131124_S38	0.17	0.16	-0.02	-10.36
R_66G59_TK_soil_131124_S39	0.14	0.12	-0.02	-14.02
R_67H59_TK_soil_131124_S40	0.18	0.16	-0.02	-12.29
R_6H610_TK_soil_131124_S238	0.25	0.23	-0.01	-6.00
R_70B69_TK_soil_131124_S42	0.24	0.23	-0.01	-4.46
R_71C69_TK_soil_131124_S43	0.22	0.20	-0.02	-8.87
R_75D69_TK_soil_131124_S44	0.30	0.28	-0.02	-5.71
R_76E69_TK_soil_131124_S45	0.17	0.15	-0.03	-14.75
R_79G69_TK_soil_131124_S47	0.26	0.25	-0.01	-4.91
R_80H69_TK_soil_131124_S48	0.29	0.26	-0.03	-10.13
R_87F79_TK_soil_131124_S54	0.23	0.18	-0.05	-21.72
R_88G79_TK_soil_131124_S55	0.18	0.16	-0.02	-10.86
R_8A710_TK_soil_131124_S239	0.22	0.21	-0.01	-6.11
R_91A89_TK_soil_131124_S57	0.20	0.18	-0.02	-9.38
R_92B89_TK_soil_131124_S58	0.15	0.12	-0.03	-17.79
R_94D89_TK_soil_131124_S60	0.15	0.12	-0.02	-15.62
R_96E89_TK_soil_131124_S61	0.18	0.16	-0.02	-10.18
R_97F89_TK_soil_131124_S62	0.19	0.17	-0.02	-9.15
R_98G89_TK_soil_131124_S63	0.18	0.14	-0.04	-20.57
R_99H89_TK_soil_131124_S64	0.28	0.26	-0.02	-7.55
R_245F211_TK_root_131124_S18	0.19	0.17	-0.03	-14.58
R_9B710_TK_soil_131124_S240	0.19	0.15	-0.05	-24.45
R_120E119_TK_soil_131124_S85	0.14	0.13	-0.02	-10.54
R_173E510_TK_soil_131124_S187	0.22	0.19	-0.03	-13.64
R_22E19_TK_soil_131124_S5	0.19	0.16	-0.03	-13.86
R_286A711_TK_root_131124_S141	0.13	0.12	-0.01	-8.31
R_292F711_TK_root_131124_S146	0.26	0.16	-0.10	-37.42
R_293G711_TK_root_131124_S147	0.19	0.17	-0.02	-10.74
R_294H711_TK_root_131124_S148	0.11	0.11	-0.01	-6.55
R_31C29_TK_soil_131124_S11	0.19	0.17	-0.03	-13.25
R_37G29_TK_soil_131124_S15	0.14	0.13	-0.02	-11.05
R_383D1210_TK_root_131124	0.25	0.22	-0.03	-13.67
R_108A109_TK_soil_131124	0.21	0.17	-0.04	-19.65
R_302H811_TK_root_131124_S156	0.22	0.18	-0.03	-15.86

Table S6. Statistics for exudation rate and soil respiration using linear mixed effect model and emmeans.

		F	Df	Df.res	Pr(>F)
exudation rate	(Intercept)	124.54	1	16.57	4.02E-09
	season	37.04	1	190.27	6.25E-09
	treatment	0.01	1	16.57	0.943
	species	5.67	2	15.13	0.015
	season:treatment	0.67	1	190.27	0.416
	season:species	0.63	2	190.13	0.535
	treatment:species	0.89	2	15.13	0.431
	season:treatment:species	0.95	2	190.13	0.388

	contrast - emmeans	species	estimate	SE	df	t.ratio	p.value
	dry season - wet season	*Pinus*	27.49	5.48	187.81	5.02	1.19E-06
	dry season - wet season	*Pistacia*	17.18	7.76	190.81	2.21	0.028017
	dry season - wet season	*Quercus*	27.44	7.13	190.49	3.85	0.000161

		F	Df	Df.res	Pr(>F)
soil respiration	(Intercept)	41.11	1	6	0.001
	season	34.11	1	6	0.001
	treatment	0.18	1	6	0.685
	species	3.31	2	6	0.108
	season:treatment	1.87	1	6	0.221
	season:species	2.75	2	6	0.142
	treatment:species	2.14	2	6	0.199
	season:treatment:species	3.60	2	6	0.094

	contrast - emmeans	species	estimate	SE	df	t.ratio	p.value
	dry season - wet season	*Pinus*	-5.86	1.27	6	-4.61	0.004
	dry season - wet season	*Pistacia*	-1.89	1.27	6	-1.49	0.187
	dry season - wet season	*Quercus*	-5.10	1.27	6	-4.02	0.007

Table S7. Results for Permanova of Metabolites including all factors: tree species, rain-exclusion treatment (treatment), time, and their interaction effects (Analysis: overall); and Results for Permanova of Metabolites for each tree species with factors: treatment, time and their interaction effects (Analysis: *Pinus, Pistacia, and Quercus*).

Analysis	Term	Df	SumOfSqs	R^2	F	Pr(>F)
overall	**species**	2	10364.080	0.195	12.735	1e-04
overall	**treatment**	1	424.144	0.008	1.042	1e-04
overall	**time**	3	14741.756	0.278	12.076	1e-04
overall	**species:treatment**	2	878.788	0.017	1.080	1e-04
overall	**species:time**	6	6850.873	0.129	2.806	0.005
overall	treatment:time	3	913.274	0.017	0.748	0.691
overall	species:treatment:time	6	1815.185	0.034	0.743	0.768
overall	Residual	42	17090.186	0.322	NA	NA
overall	Total	65	53078.284	1.000	NA	NA
Pinus	**treatment**	1	632.423	0.036	1.396	0.003
Pinus	time	3	8626.487	0.495	6.345	3e-04
Pinus	treatment:time	3	911.077	0.052	0.670	0.744
Pinus	Residual	16	7250.498	0.416	NA	NA
Pinus	Total	23	17420.484	1.000	NA	NA
Pistacia	**treatment**	1	221.771	0.013	0.631	3e-04
Pistacia	**time**	3	10072.143	0.612	9.556	1e-04
Pistacia	treatment:time	3	890.940	0.054	0.845	0.599
Pistacia	Residual	15	5270.061	0.320	NA	NA
Pistacia	Total	22	16454.915	1.000	NA	NA
Quercus	**treatment**	1	418.554	0.047	1.008	0.049
Quercus	**time**	3	2924.182	0.331	2.346	0.006
Quercus	treatment:time	3	926.442	0.105	0.743	0.773
Quercus	Residual	11	4569.628	0.517	NA	NA
Quercus	Total	18	8838.806	1.000	NA	NA

Table S8. Results for Permanova of Microbiome including all factors: root/ soil samples (sample_type), tree species, rain- exclusion treatment (treatment), time, and their interaction effects (Analysis: Overall); and Results for Permanova of Microbiome for root and soil separately with factors: species, treatment, time and their interaction effects (Analysis: Soil and Root).

Analysis	Term	Df	SumOfSqs	R^2	F	Pr(>F)
Overall	**sample_type**	1	6.597	0.117	23.198	0.001
Overall	**specie**	2	2.367	0.042	4.162	0.001
Overall	**treatment**	1	0.533	0.009	1.876	0.020
Overall	time	5	1.405	0.025	0.988	0.485
Overall	**sample_type:specie**	2	1.752	0.031	3.081	0.001
Overall	**sample_type:treatment**	1	0.749	0.013	2.632	0.002
Overall	specie:treatment	2	0.663	0.012	1.166	0.204
Overall	sample_type:time	5	1.415	0.025	0.995	0.460
Overall	specie:time	10	2.382	0.042	0.838	0.961
Overall	treatment:time	5	1.197	0.021	0.841	0.881
Overall	sample_type:specie:treatment	2	0.738	0.013	1.297	0.102
Overall	sample_type:specie:time	10	2.672	0.048	0.939	0.694
Overall	sample_type:treatment:time	5	1.322	0.024	0.930	0.670
Overall	specie:treatment:time	10	2.106	0.037	0.741	0.999
Overall	sample_type:specie:treatment:time	6	1.573	0.028	0.922	0.718
Overall	Residual	101	28.724	0.511	NA	NA
Overall	Total	168	56.197	1.000	NA	NA
Soil	**specie**	2	1.622	0.070	3.053	0.001
Soil	treatment	1	0.390	0.017	1.469	0.108
Soil	time	5	1.134	0.049	0.854	0.782
Soil	specie:treatment	2	0.554	0.024	1.043	0.363
Soil	specie:time	10	1.693	0.073	0.637	1.000
Soil	treatment:time	5	1.060	0.046	0.798	0.880
Soil	specie:treatment:time	10	1.877	0.081	0.707	0.997
Soil	Residual	56	14.876	0.641	NA	NA
Soil	Total	91	23.206	1.000	NA	NA
Root	**specie**	2	2.517	0.095	4.090	0.001
Root	**treatment**	1	0.884	0.034	2.874	0.001
Root	time	5	1.667	0.063	1.084	0.247
Root	specie:treatment	2	0.829	0.031	1.347	0.052
Root	specie:time	10	3.379	0.128	1.098	0.157
Root	treatment:time	5	1.466	0.056	0.953	0.634
Root	specie:treatment:time	6	1.802	0.068	0.976	0.559
Root	Residual	45	13.848	0.525	NA	NA
Root	Total	76	26.393	1.000	NA	NA

Table S9. Results for Linear Discriminant Analysis Effect Size (LEfSe) of root microbiome with LDA scores for (A) tree species *Pinus* (Ph), *Pistacia* (Pl) and *Quercus* (Qc) and (B) seasons.

A

	Pvalues	FDR	Ph	Pl	Qc	LDAscore
Mycobacterium	0.001	0.016	52560.00	6293.70	52902.00	4.37
Actinomadura	0.003	0.031	15792.00	1752.20	12568.00	3.85
uncultured Blastocatellia_11_24	0.001	0.012	765.23	10.48	13546.00	3.83
uncultured Sandaracinaceae	0.000	0.000	55.58	11691.00	722.62	3.76
Rhizobacter	0.026	0.130	15308.00	17293.00	6678.30	3.72
Actinoplanes	0.004	0.031	17079.00	20226.00	10130.00	3.70
uncultured Chitinophagaceae	0.001	0.012	9332.20	764.80	12.04	3.67
Amycolatopsis	0.002	0.023	2898.00	8548.00	92.89	3.63
Phenylobacterium	0.000	0.000	6762.50	2394.20	257.46	3.51
Microvirga	0.022	0.124	7276.70	10636.00	4122.10	3.51
Massilia	0.021	0.124	7269.10	7206.20	1101.50	3.49
Ensifer	0.000	0.001	5991.80	167.44	1.00	3.48
uncultured Microtrichales	0.001	0.011	4103.90	861.12	42.58	3.31
uncultured Polyangiales_Blrii41	0.030	0.139	4187.80	6183.30	3187.30	3.18
Ideonella	0.001	0.011	2620.00	333.56	226.38	3.08
Lysobacter	0.005	0.039	2196.40	1276.90	1.00	3.04
Duganella	0.012	0.086	1825.80	379.28	217.12	2.91
Thermoanaerobaculaceae_Subgroup10	0.001	0.012	330.35	1789.20	1683.50	2.86
Blastococcus	0.002	0.023	119.12	1284.20	1.00	2.81
Edaphobacter	0.040	0.175	1059.60	1.00	23.92	2.72
uncultured Rubrobacteria_67_14	0.019	0.119	1056.20	1557.60	560.27	2.70
Alterococcus	0.000	0.004	318.81	698.80	1.00	2.54
Altererythrobacter	0.021	0.124	15.77	611.32	228.88	2.48
uncultured Methyloligellaceae	0.000	0.011	16.50	77.00	500.42	2.39
Sphingomonadaceae_Ellin6055	0.030	0.139	89.92	535.84	565.85	2.38
Vicinamibacterales_ASV_213779	0.039	0.172	41.04	471.32	343.08	2.33
Geodermatophilus	0.003	0.030	54.08	467.00	59.73	2.32
uncultured Reyranellaceae	0.014	0.094	61.69	1.00	414.38	2.32
Brevundimonas	0.030	0.139	500.54	153.56	169.69	2.24
uncultured Armatimonadales	0.000	0.011	127.19	382.04	49.35	2.22
uncultured Roseiflexaceae	0.023	0.125	76.77	366.72	249.69	2.16
Methylobacterium_Methylorubrum	0.014	0.094	218.92	464.52	314.38	2.09
Paenibacillus	0.005	0.039	237.19	18.88	21.81	2.04

B

	Pvalues	FDR	April_22	April_23	Dec_22	July_23	June_22	Sept_22	LDAscore
Cutibacterium	0.005	0.52	1937.70	22.94	235.50	17.83	104.75	110.33	2.98
Actinoallomurus	0.008	0.52	500.17	2463.80	1.00	762.33	197.75	1.00	3.09
Chitinophaga	0.012	0.52	8395.50	127.12	3225.20	15.00	96.00	1957.70	3.62
Brevundimonas	0.018	0.57	979.92	11.25	287.81	1.00	61.58	265.07	2.69
Ideonella	0.022	0.57	1711.60	6.50	1027.20	1.00	1836.20	1548.30	2.96
Pseudomonas	0.029	0.57	52011.00	272.50	6476.20	900.17	1492.10	3664.10	4.41
Candidatus_Nitrocosmicus	0.037	0.57	93.58	607.38	196.12	149.17	8.08	83.07	2.48
Massilia	0.038	0.57	18192.00	812.94	7343.50	1421.30	879.83	1992.90	3.94

Supplementary Figures

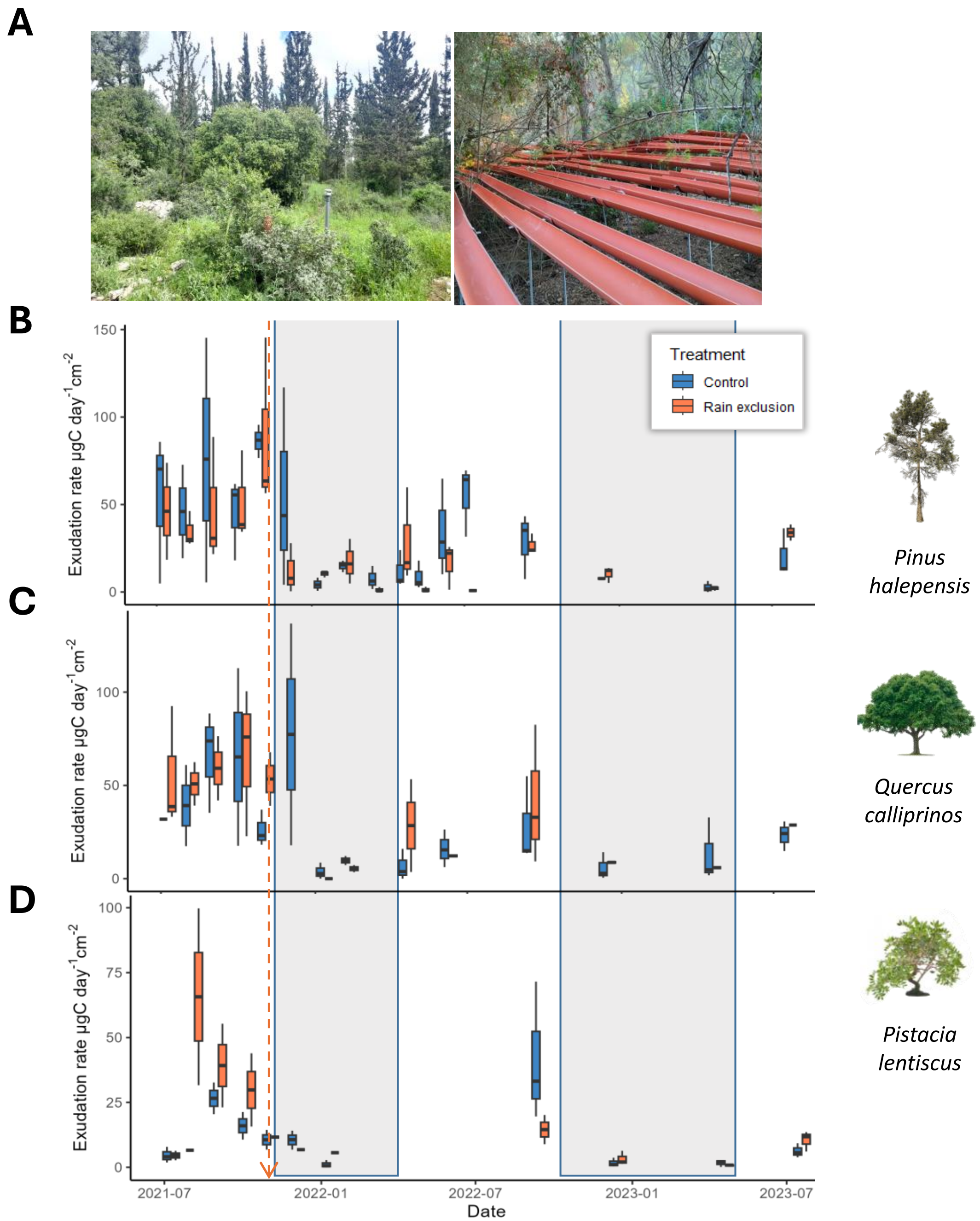

Figure S1. Three control plots (left) and three rain-exclusion plots (right) located in the mixed Mediterranean forest study site (A). Seasonal root exudation rates (µg C day^{-1} cm^{-2}) in control (blue) and rain-exclusion (orange) plots from July 2021 to July 2023 of *Pinus* (B), *Quercus* (C) and *Pistacia* (D). Dashed orange line marks the establishment of the rain exclusion in November 2021.

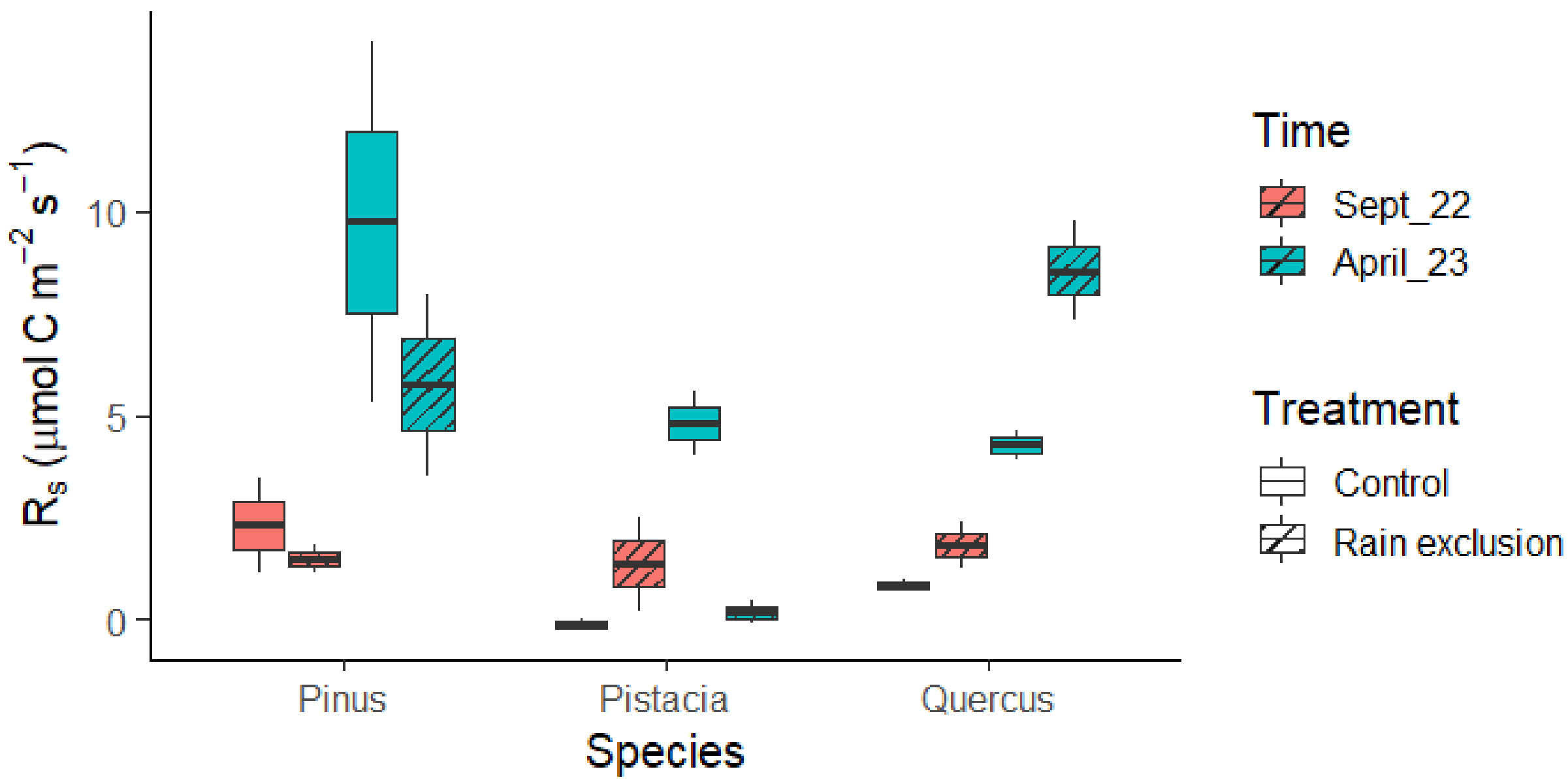

Figure S2. Soil respiration (Rs) measured beneath *Pinus, Pistacia and Quercus* in Mediterranean forest in control and in rain exclusion plots in the dry season (Sept_22) and wet season (April_23; n=2).

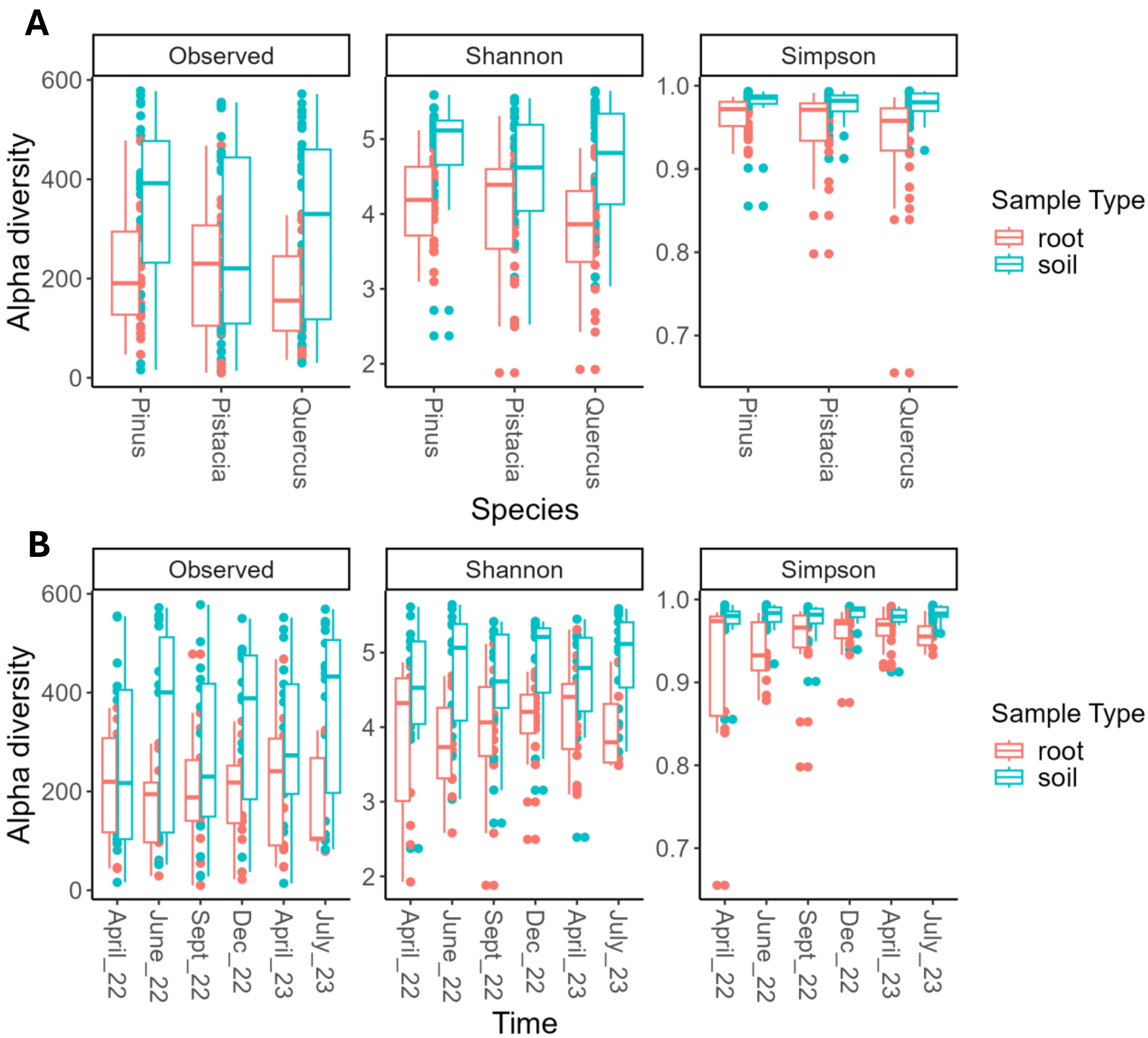

Figure S3. Alpha diversity (Observed ASVs, Shannon-Wiener index, Simpson's diversity index) among rhizosphere and soil microbial community of different species (A) and time (B).

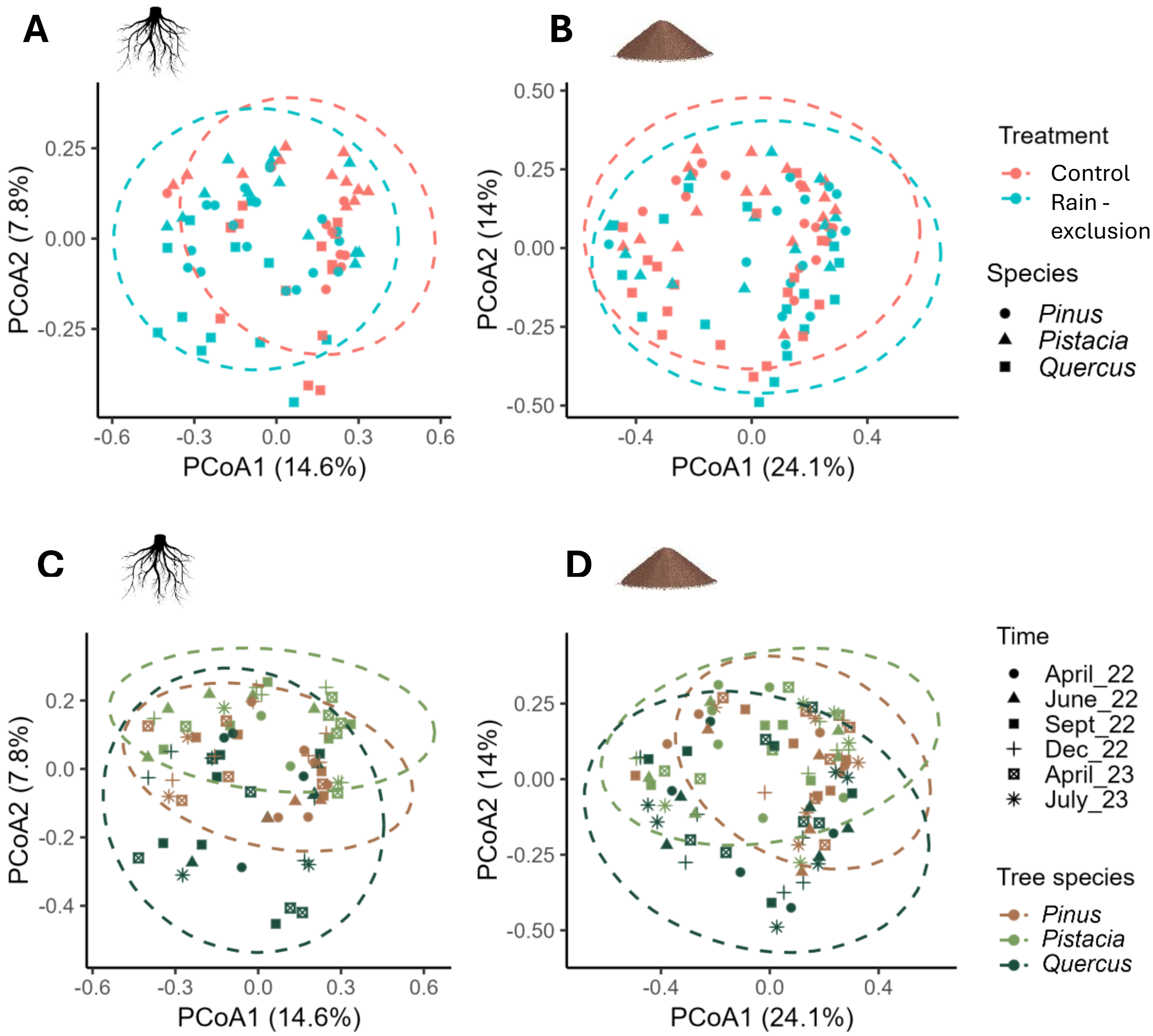

Figure S4. Principal coordinates analysis (PCoA) of bacterial communities based on Bray-Curtis distance at the ASV level for rhizosphere (A, C) and soil community (B, D) clustered by treatment (A, B) and species (C, D).

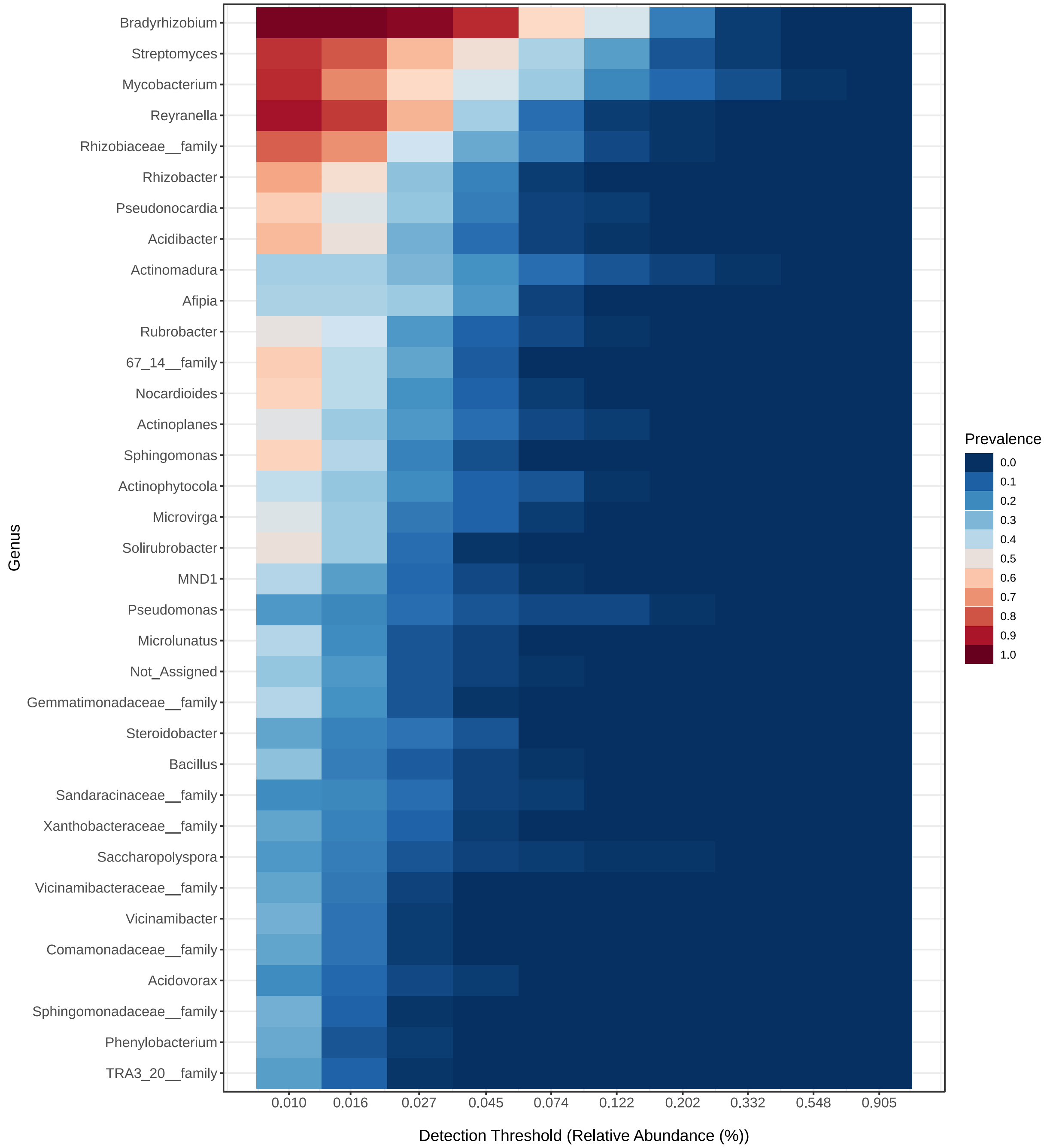

Figure S5. Genera of core microbiome in the rhizosphere.

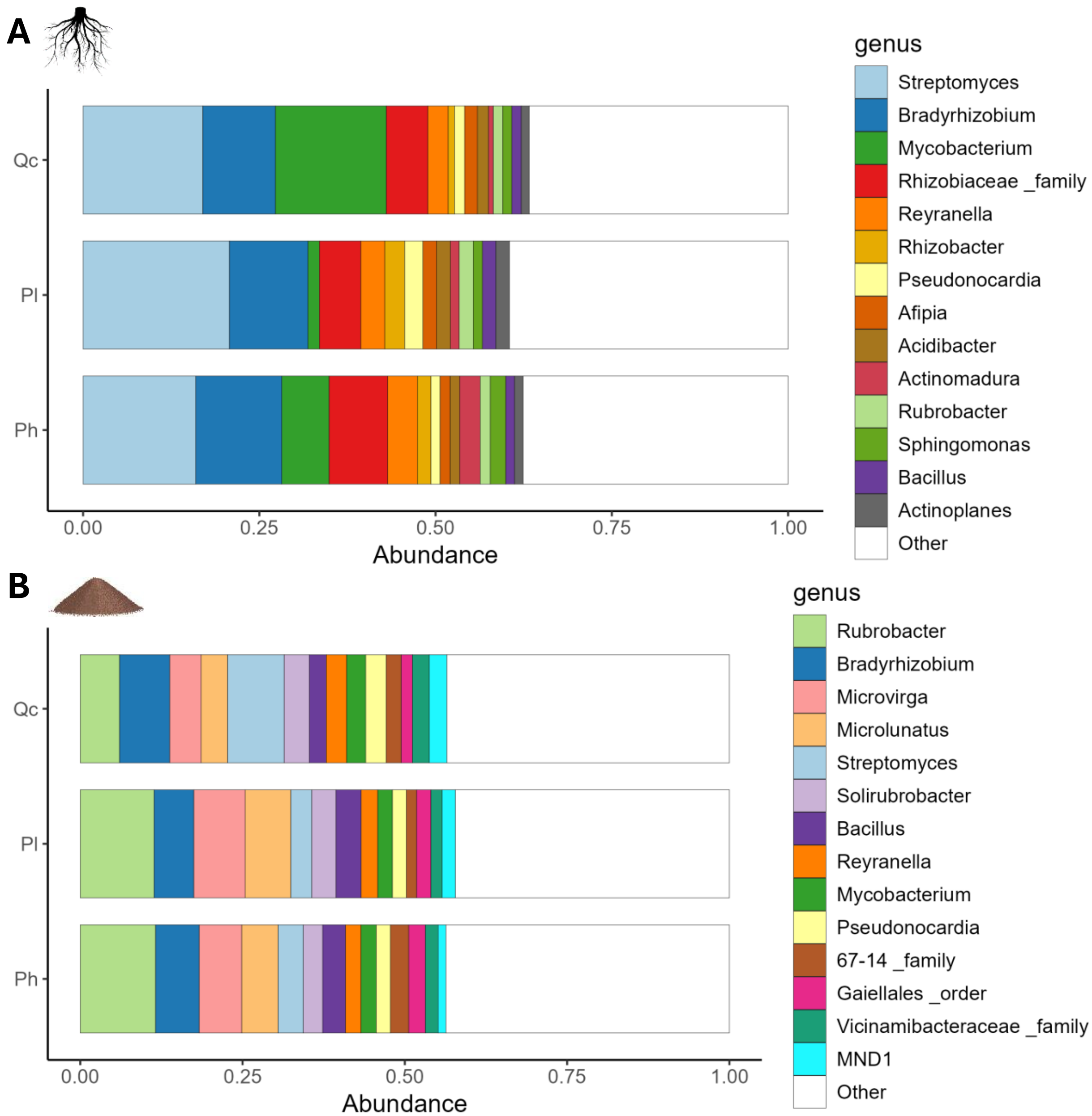

Figure S6. Abundance of Genera of bacteria in root (A) and soil (B) for *Pinus* (Ph), *Pistacia* (Pl) and *Quercus* (Qc).

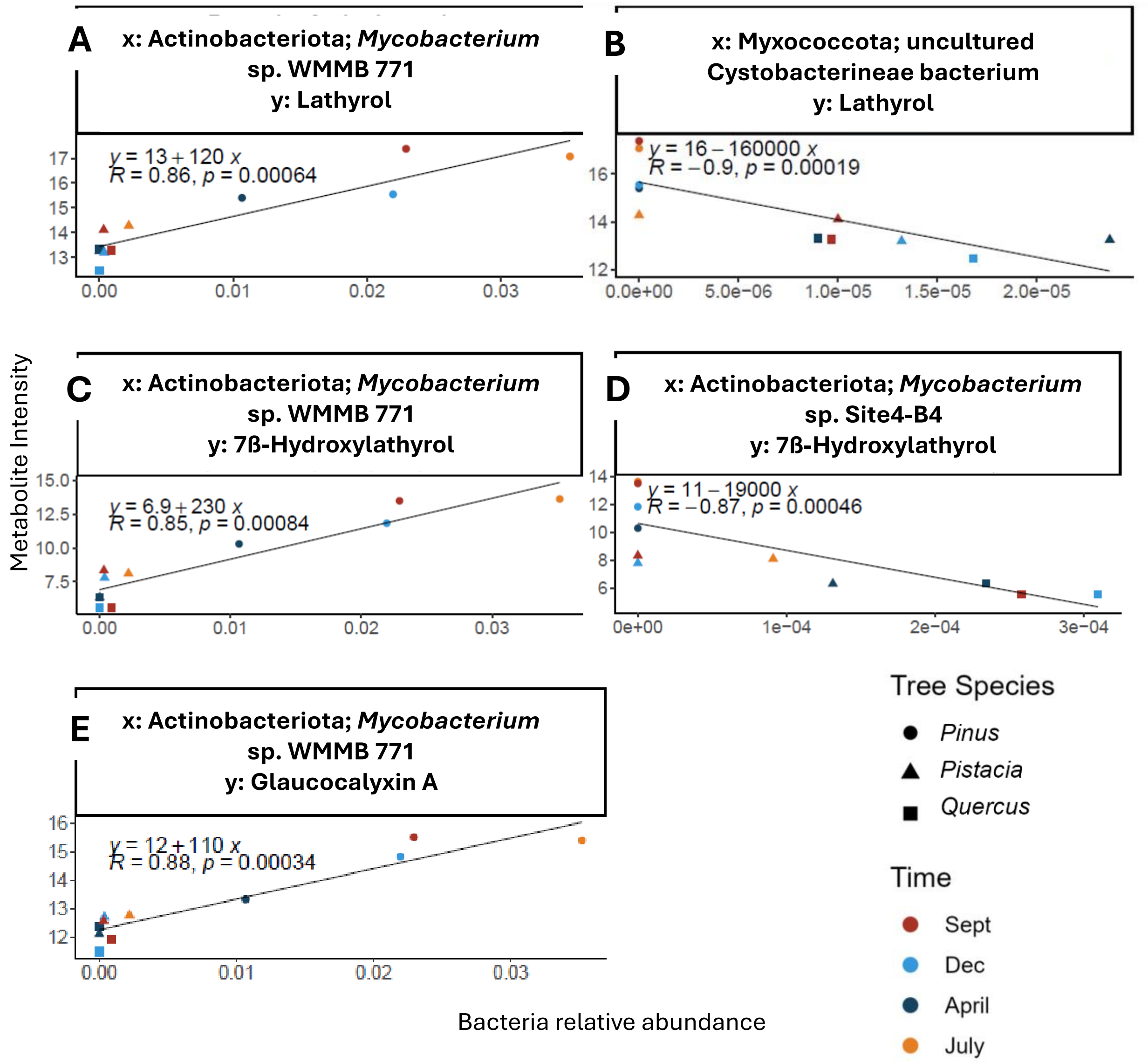

Figure S7. Metabolite–microbe associations for selected correlations from Fig. 6 across tree species *Pinus, Pistacia and Quercus* and seasons (Sept 22, Dec 22, April 23 and July 23). Panels A–E show example metabolite–microbe associations. Regression line shows the OLS fit with equation, Spearman's ρ, and p-value.
